# Psychometric validation of the Hypoparathyroidism Patient Experience Scales (HPES)

**DOI:** 10.1186/s41687-021-00320-2

**Published:** 2021-08-10

**Authors:** Meryl Brod, Lori McLeod, Denka Markova, Jill Gianettoni, Sanchita Mourya, Zhengning Lin, Aimee Shu, Alden Smith

**Affiliations:** 1 The Brod Group, 219 Julia Ave, CA 94941 Mill Valley, USA; 2 RTI Health Solutions (RTI International), 3040 East Cornwallis Road, Research Triangle Park, NC 27709 USA; 3 Ascendis Pharma, 500 Emerson Street, Palo Alto, CA 94301 USA

**Keywords:** Hypoparathyroidism, Psychometrics, Quality of life, Patient-reported outcome measures, Surveys and questionnaires, Adult

## Abstract

**Background:**

Hypoparathyroidism (HP) is a rare endocrine disorder characterized by absent or inappropriately low levels of circulating parathyroid hormone with associated significant physical and cognitive symptoms. This study evaluated the psychometric properties of the Hypoparathyroidism Patient Experience Scales (HPES), which were developed as disease-specific, patient-reported outcome (PRO) measures to assess the symptoms and impacts associated with HP in adults.

**Methods:**

Data from a non-interventional, observational study (*N* = 300) and a Phase 2 clinical trial (*N* = 59) were used in the psychometric evaluation. Observational and trial assessments included: an online validation battery (baseline or screening) and retest (approximately 2 weeks after baseline or screening). In the trial, the primary efficacy endpoint was assessed at week 4 through re-administration of the HPES and validation battery subset. The observational study’s larger sample size allowed for evaluation of the HPES descriptive properties, scoring algorithm, test-retest reliability, and construct validity. The trial data examined responsiveness, meaningful within-patient change estimates, and treatment impact on HPES scores.

**Results:**

Demographic and self-reported medical characteristics results were similar across the 2 studies. Factor analysis confirmed domains in the HPES-Symptom (*n* = 2) and HPES-Impact (*n* = 4). For both measures, total and domain scores demonstrated acceptable reliability and validity for both the observational and trial samples. Internal consistency evidence was strong. Test-retest reliability estimates generally approached the recommended 0.70 threshold. The construct validity correlations with other PRO measures were mainly as hypothesized, thus supporting the HPES scores and constructs. Mean scores for both measures also differed as anticipated and significantly across known-groups, thus providing evidence for the scores discriminating between meaningful groups. Trial results supported both HPES total and domain scores’ ability to detect change. The difference in mean total and domain scores for both measures demonstrated statistically significant improvements for TransCon PTH compared to placebo treated subjects despite the small sample and a short 4-week duration on fixed, non-optimized doses.

**Conclusions:**

The HPES were found to be conceptually sound with adequate evidence supporting their reliability and validity. Incorporation of the HPES into clinical and research settings will help to further elucidate and assess the patient experience of living with HP and identify treatment differences.

**Supplementary Information:**

The online version contains supplementary material available at 10.1186/s41687-021-00320-2.

## Background

Hypoparathyroidism (HP) is a rare endocrine disorder characterized by absent or inappropriately low levels of circulating parathyroid hormone (PTH) [1, 2]. Low levels or the absence of PTH circulating in the bloodstream can lead to hypocalcemia, hyperphosphatemia, hypercalciuria, and overly-mineralized bone [1, 3]. This condition most commonly results from neck surgery, but may also be inherited, associated with other disorders, or idiopathic in its etiology [2, 4]. HP is typically treated with oral calcium and vitamin D supplements [1–3, 5]. PTH [1–84] replacement therapy has been approved by the United States (U.S.) Food & Drug Administration (FDA) and the European Medicines Agency for adults who do not respond to standard of care (SOC) (active vitamin D and calcium supplements) [1, 6].

Significant physical and cognitive symptoms are associated with HP including fatigue, muscle cramping/spasms, paresthesia, cognitive dysfunction, and sleep disturbances [1, 7–12]. Previous research has demonstrated that patients with HP more frequently report experiencing these symptoms compared with either the general population [7, 8] or matched case controls [9, 10]. Many patients have further reported experiencing symptoms associated with HP despite being on SOC and/or PTH replacement therapy [7, 8, 11, 12]. Research also indicates that patients with HP on SOC and/or PTH replacement therapy may have a reduced health-related quality of life (HRQOL), experiencing a range of impacts including anxiety, depression, and interference with daily life and work productivity [1, 7, 8, 10, 12–19] when compared with the normative reference range and controls [8, 9, 16, 20]. In a web-based survey of 374 adults with HP in the USA, 45% reported significant interference with their life due to HP, and 20% attributed a change in their work status to HP symptoms, including switching from full-time to part-time employment, becoming unemployed, going on disability, or retiring [11]. A study of patients with HP in Norway, in turn, found that 40% reported receiving either permanent or temporary social security benefits compared with only 14% of the country’s general adult population [16].

Although previous research has provided evidence that HRQOL may be reduced in patients with HP, the validated questionnaires used in most studies were not disease-specific and did not assess a number of symptoms associated with this condition, such as cognitive deficits, fatigue, or decreased muscle strength [13, 21]. Additionally, prior disease-specific measures that have been developed for this condition have focused primarily on symptoms and have not captured the broad spectrum of both symptom and disease impacts [22, 23].

To address this gap, the Hypoparathyroidism Patient Experience Scales (HPES) were developed as disease-specific, patient-reported outcome (PRO) measures to assess the symptoms and impacts associated with HP in adults: the 17-item HPES-Symptom and the 26-item HPES-Impact. Conceptual development of these scales has previously been reported [24, 25] and was based on the scientific principles outlined in the FDA PRO guidance [26] and best practices for PRO measure development [27–30], including reviews of the HP literature, interviews with clinical experts, and direct patient input through qualitative concept elicitation and cognitive debriefing interviews with a combined total of 58 adults in the USA. The hypothesized domains covered by the HPES-Symptom are physical and cognitive signs and symptoms of HP. The hypothesized domains of the HPES-Impact include HP impact on physical functioning, daily life, psychological well-being, and social life and relationships.

The purpose of this study was to evaluate the HPES in order to assess the measurement model and psychometric properties of the measures to determine the validity, reliability, sensitivity to change, and interpretability of the HPES in the intended patient population.

## Methods

### Study design

Data from the following two sources were used in the psychometric evaluation of the HPES: a non-interventional, observational study and a Phase 2 clinical trial. The observational study provided a larger sample size, which allowed for a robust evaluation of the HPES measures’ descriptive properties, scoring algorithm, test-retest reliability, and construct validity (based on correlations and known-groups validity).

The Phase 2 trial provided an opportunity to confirm the reliability and validity findings based on the observational study data and to extend the evaluation to include longitudinal properties such as responsiveness (ability to detect change) as well as initial estimates of meaningful within-patient change. Additionally, the trial data provided the opportunity to examine the impact of treatment on the HPES scores.

#### Non-interventional, observational study design

In the non-interventional, observational study, a PRO validation battery, including all measures needed to conduct the psychometric validation, was administered online to a sample of 300 adults with HP residing in the USA at a single time point (baseline). All participants were invited to complete an online retest approximately 2 weeks after baseline to facilitate the evaluation of test-retest reliability of the HPES. The retest included the HPES and two dichotomous questions regarding major life events (yes/no) and HP treatment changes (yes/no).

The study was approved by an independent Institutional Review Board (IRB), Copernicus Group IRB (tracking #20190783), located in Cary, North Carolina, USA. Informed consent was obtained from all participants.

#### Phase 2 clinical trial design

The PaTH Forward clinical trial (TransCon PTH TCP-201, ClinicalTrials.gov identifier NCT04009291) is a Phase 2, multicenter, randomized, double-blind, placebo-controlled, parallel group trial with an open-label extension, investigating the safety, tolerability and efficacy of TransCon PTH, an investigational once-daily long-acting prodrug of parathyroid hormone administered subcutaneously daily in adults with HP. The ongoing PaTH Forward trial included a screening period (4-weeks), a randomized, double-blind, placebo-controlled period (4-weeks) followed by an open-label extension period (210-weeks). Subjects were randomized 1:1:1:1 to four arms: TransCon PTH at one of three fixed doses along with active vitamin D and calcium; and placebo, co-administered with active vitamin D and calcium. The primary efficacy endpoint was assessed at Week 4 (the end of the double-blind, fixed dose period). Only data from the 4-week blinded treatment period were used for the validation study. Participants were recruited from six countries (Germany, Denmark, Italy, Norway, Canada, and USA).

The same PRO validation battery was administered to subjects at screening, and retest administered at trial visit 1 (week 0) (approximately 2 weeks from screening), as administered in the observational study. In addition, the HPES and a subset of the validation battery were re-administered at trial visit 3 (week 4).

Ethics approvals for the participating clinical trial study sites were obtained in all countries (detailed in ethics approval and consent to participate statement). Informed consent was obtained from all participants.

#### Inclusion/exclusion criteria

Key inclusion criteria for the observational study and Phase 2 trial were: (1) males and females aged ≥18 years, (2) with a diagnosis of HP  ≥6 months (post-surgical, autoimmune, genetic, or idiopathic); in addition, (3) observational study participants had stable HP for at least 3 months (infrequent severe hypo- or hypercalcemia [low or high calcium levels] not more than two or three times a week), and (4) Phase 2 trial subjects were on a stable dose of SOC, had optimization of supplements to have all subjects achieve serum calcium within lower half of the normal range before randomization, and had thyroid-stimulating hormone within normal lab limits.

Key exclusion criteria for both studies were: (1) known activating mutation in the Calcium-Sensing Receptor (*CASR*) gene; (2) impaired responsiveness to PTH (pseudohypoparathyroidism); and (3) having other disease that might affect calcium metabolism or calcium-phosphate homeostasis or PTH levels.

### Measures

In addition to the HPES, the PRO validation battery included sociodemographic items, questions on participants’ HP medical history, Patient Global Impression of Severity (PGIS; with response categories - no noticeable symptoms, very mild, mild, moderate, severe, very severe), and study-specific resource utilization questions. The battery also included the Multidimensional Fatigue Inventory (MFI) [31] using an altered recall period of “past 2 weeks” from the original of “lately” with the permission of the developer, the post-external malaise and sleep disruptions domains of the DePaul Symptom Questionnaire (DSQ-2) [32], Cognitive Failures Questionnaire (CFQ) [33], SF-36v2 [34, 35], Sheehan Disability Scale (SDS) [36], MOS Social Support Survey (MOS-SSS) [37], and Hospital Anxiety and Depression Scale (HADS) [38].

For the Phase 2 study, clinicians also completed Clinician Global Impression of Severity (CGIS) items at screening, visits 1 (week 0), and 3 (week 4). In addition, biomarkers (serum and urine calcium levels) and information on supplement intake collected from the Phase 2 study were used in the psychometric evaluation.

### Statistical analysis methods

All analyses were conducted following an a-priori psychometric analysis plan. All statistical tests used a significance level of 0.05 (two-sided) unless otherwise noted and were applied to identify patterns. Statistics were conducted using SAS [39].

#### Sociodemographic and medical characteristics

Descriptive statistics were calculated for demographic and self-reported medical variables to describe the study sample.

#### Descriptive item measurement characteristics

Descriptive statistics were calculated for the item-level, domain-level and total scores of the HPES-Symptom and HPES-Impact. The floor/ceiling effects threshold for closer examination was set at 40% for endorsement of the extreme response categories (e.g., 0: Never, Not at all; 4: Very Often/Always, Extremely).

#### Item reduction

Items were considered for deletion for reasons of high correlations with other items, floor or ceiling effects, and poor fit to the factor analysis model. Item-to-item correlation was examined by a correlation matrix of each item in the HPES–Symptom and HPES–Impact. Possible redundancy was flagged for pairs of items with high inter-item polychoric correlation coefficients (|r| > 0.80) and pairs of items with low inter-item correlations (|r| < 0.30). The complete correlation matrix, the factor structure, and qualitative results were also used to make decisions regarding redundancy.

Item-to-total correlations were examined for every item used to form HPES-Symptom and HPES-Impact domain scores; correlation coefficients of at least 0.40 were considered adequate.

#### Factor analyses

A confirmatory factor analysis (CFA) was conducted using polychoric correlations and weighted least squares estimation to verify the final factor structure by separately analyzing the HPES-Symptom baseline item-level data and the HPES-Impact baseline item-level data from the observational study only, due to the small Phase 2 trial sample size. Criteria for CFA model fit included the root mean square error of approximation (RMSEA), standardized root mean squared error (SRMR), comparative fit index (CFI) [40] and Tucker-Lewis Index (TLI) [41]. The following values are desirable for these indices: RMSEA < 0.10; SRMR < 0.08, CFI > 0.95 and TLI > 0.95 [42].

#### Test-retest reliability

To evaluate the test-retest reliability, or stability, of the HPES-Symptom and HPES-Impact finalized scores (domain and total) intraclass correlation coefficients (ICCs) based on a two-way (subjects × time) mixed-effects analysis of variance (ANOVA) with absolute agreement were computed. Data were used from two consecutive time points – baseline and approximately 2 weeks retest in the observational study and screening and visit 1 (week 0) (approximately 2 weeks from screening) in the Phase 2 trial – for the following groups of subjects: overall; no change in major life events; and no change in treatment [43]. It is generally recommended that ICCs be at least 0.70 for multi-item scales [44].

Internal consistencies of the proposed HPES-Symptom and HPES-Impact domain scores using Cronbach’s coefficient alphas [45] were computed.^1^ The approximate range of optimal alphas [46] is between 0.70 and 0.90, indicating a set of items that is strongly related and capable of supporting a unidimensional scoring structure but not redundant [46].

#### Construct validity

Correlational analyses were conducted, according to a-priori hypotheses, to examine the construct validity of the HPES finalized scores (subdomain, domain, and total) by study, using data from baseline in the observational study and data from screening and visit 3 (week 4) in the Phase 2 trial.

The magnitude and direction of the resulting Pearson correlation coefficients were compared with respect to specific a-priori hypotheses and to Cohen’s [47] guideline for interpreting correlation coefficients: absolute values of correlations of 0.50 or greater are considered strong, correlations that fall between 0.30 and 0.49 are moderate, and those that fall between 0.10 and 0.29 are small. Overall, the strength of correlations between specific HPES-Symptom and HPES-Impact domain scores and supporting measures that assess similar content was hypothesized to be at least moderate (|r| > 0.30) and stronger than with measures that assess different contents. The a priori hypotheses for the correlations are presented in Table 3 and Table 8 in the Results section.

#### Known-groups validity

To evaluate the ability of the HPES to distinguish between groups that are hypothesized to differ, known-groups validity was assessed using ANOVA for each domain and the total score based on a-priori hypotheses using a two-tailed test at a *p* < 0.05 level using data from baseline in the observational study and data from screening and visit 3 (week 4) in the Phase 2 trial. The a priori hypotheses for the known-groups validity evaluations are presented in Table 4 and Table 9 in the Results section.

#### Ability to detect change

Ability to detect change, or responsiveness, refers to the extent to which an instrument can detect changes in patients who have changed in clinical status [48]. Mean differences in the HPES change scores (screening to visit 3) were compared across levels of external criteria characterizing change using paired t-tests or ANOVA. Responsiveness of the HPES-Symptom and HPES-Impact scores (domains and total) were also assessed by reviewing correlations between these HPES change scores and changes in the supporting measures used to support construct validity using change scores. The a priori hypotheses for the responsiveness evaluations are presented in the Results sections under each measure.

#### Threshold for meaningful within-patient change (responder definition)

To identify patients who experienced a meaningful improvement in their symptoms and impacts over the course of treatment, a preliminary responder threshold (responder definition) was determined to characterize a meaningful within-patient change in the scores of the PRO measure. Patients were classified as achieving a meaningful within-patient improvement (or responder) using the optimal anchor measure and a proposed anchor criterion (e.g., a 1-point improvement in PGIS). Mean change in the HPES scores (from screening to visit 3) for the subgroup of patients achieving the anchor criterion was proposed as the primary estimate for a responder threshold characterizing a meaningful within-patient change. The median change was proposed as a supportive estimate and used to evaluate the skewness of the distribution.

In addition to meaningful within-patient change thresholds estimated using anchor-based methods, two commonly applied distribution-based methods, the half-standard deviation and standard error of measurement were examined. Distribution-based estimates are often viewed by PRO experts as a lower-bound for estimating meaningful within-patient change. For these computations, baseline standard deviations (SD) and the lowest test-retest ICC were used [49].

Treatment comparison between TransCon PTH and placebo at week 4 for HPES was conducted based on a pre-planned analysis of covariance (ANCOVA) with baseline score as the covariate, and treatment assignment as a fixed factor.

## Results

### Sample description

Overall, the demographic and self-reported medical characteristics results were similar across the observational and Phase 2 studies. For the 300 observational study participants, 196 (65.3%) were female. The mean age of the sample was 44.0 years (SD = 10.5 years). The participants were predominantly married (82.7%), employed (83.3%), white (76.3%), and had a college degree or higher (65.7%). The Phase 2 trial sample included 59 subjects; 47 (81.0%) were female, the mean age of was 49.8 years (SD = 12.1 years), predominately married (67.8%), employed (67.3%), white (81.4%), and had a college degree or higher (54.2%).

Regarding their medical characteristics, participants in the observational study reported a mean of 6.1 years (SD = 8.8) since their HP diagnosis, most had post-surgical HP (95.0%) and reported taking a variety of HP medications, including PTH 1–84 (Natpara) (72.7%), calcium supplements (69.7%), and prescription vitamin D supplements (68.7%). At baseline, almost three-quarters of participants indicated that it was “somewhat” or “a lot” difficult to manage their HP and over three-quarters rated their general health as “good” or “fair”. The most frequently reported other major medical conditions were hypothyroidism (28.7%), anxiety (18.3%), chronic back pain (13.7%), depression (11.0%), obesity (10.7%), and stomach or intestinal problems (10.7%).

For the Phase 2 trial sample, the mean number of years since HP diagnosis was 11.9 years (SD = 9.5), most subjects had post-surgical HP (81.4%). At screening, approximately half of the subjects reported that it was “somewhat” or “a lot” difficult to manage their HP and approximately three-quarters of subjects rated their general health as “good” or “fair.” The most frequently reported other major medical conditions were hypothyroidism (47.5%), anxiety (20.3%), depression (16.9%), osteoarthritis (13.6%), reflux disease (11.9%), stomach or intestinal problems (11.9%), hypertension (10.2%), and chronic back pain (10.2%).

Evaluations of the HPES measures are summarized separately for the HPES–Symptom and HPES–Impact.

### HPES–Symptom measure

#### Descriptive item measurement characteristics and consideration of item reduction

For the observational study, 300 participants completed the HPES-Symptom at baseline, and a test-retest sample of 185 completed the measure again approximately 2 weeks after baseline, and for the Phase 2 trial, 59 subjects completed the measure at screening, visit 1 (week 0), and visit 3 (week 4). For both studies, the full 0–4 range (Never to Very often/Always) of item response categories was endorsed by the sample.

An examination of the item-level response distributions of HPES-Symptom items for the observational study showed no evidence of problematic ceiling effects (i.e., the best state), and for the Phase 2 trial, showed a possible ceiling effect at both screening and visit 3 for Items *Muscle spasms*, *Muscle twitching*, *Being sensitive to heat*, and *Heart problems*. Furthermore, for both studies there was no evidence of floor effects (i.e., the worst state) since the percentage of participants who reported the score indicative of the worst state did not approach 40%.

There was one high inter-item correlation pair for Items *Feeling tired* and *Low energy (r* = 0.85). Evaluation of these items using the Phase 2 trial data showed that these items remained highly correlated (*r* = 0.91) and shared similar responsiveness. Given these results, the study team reviewed the qualitative development data for these item pairs. Although participants sometimes experienced these concepts together, the qualitative data provided evidence that these concepts were considered distinct by the participants. Therefore, the decision was made to retain both items.

#### Factor analyses

To evaluate the proposed domains in the HPES-Symptom, a CFA was conducted using the baseline responses to all HPES-Symptom items and based on the hypothesized structure using the observational study data only. Key results for the HPES-Symptom are provided in Table 1 for the model allowing residual correlations among items. Model fit was generally acceptable: RMSEA = 0.097 < 0.10, CFI = 0.951 > 0.95, TLI = 0.943, and standardized root mean residual (SRMR) = 0.055 < 0.08. The inter-factor correlation was 0.67. Given the strong inter-factor correlations, a hierarchical structure was proposed to support an overall total score and two subscale scores.

**Table 1:** CFA two-factor model–factor loadings (SEs) and fit indices - baseline HPES-Symptom using observational study data

HPES-Symptom domain/item	CFA 2-factor model with residual correlated standardized estimates	
	Factor 1	Factor 2
**Physical**		
1a. Muscle cramping	0.614* (0.037)	–
1b. Muscle spasms	0.578* (0.040)	–
1c. Muscle twitching	0.609* (0.037)	–
1d. Muscle weakness	0.709* (0.031)	–
1e. Tingling WITH numbness	0.691* (0.037)	–
1f. Tingling WITHOUT numbness	0.607* (0.042)	–
1g. Pain	0.760* (0.030)	–
2a. Being sensitive to heat	0.669* (0.035)	–
2b. Feeling tired	0.853* (0.018)	–
2c. Trouble sleeping	0.755* (0.029)	–
2d. Heart problems	0.643* (0.036)	–
2e. Low energy	0.888* (0.016)	–
**Cognitive**		
3a. Remembering	–	0.893* (0.019)
3b. Finding the right words	–	0.818* (0.023)
3c. Concentrating	–	0.893* (0.018)
3d. Understanding information	–	0.762* (0.028)
3e. Thinking clearly	–	0.883* (0.019)

Note: *P* value of Chi-square test < 0.0001, RMSEA = 0.097, CFI = 0.951, TLI = 0.943, SRMR = 0.055, the residual correlation between Item 1a and Item 1b is 0.528 and between Item 1b and Item 1c is 0.417

*CFA* confirmatory factor analysis, *CFI* comparative fit index, *HPES* Hypoparathyroidism Patient Experience Scale, *RMSEA* root mean square error of approximation, *SE* standard error, *SRMR* standardized root mean residual, *TLI* Tucker-Lewis Index

* *P* < 0.05 for H0: loading = 0

Correlations among all items of the HPES-Symptom at baseline of the observational study showed that for every item, the strongest correlation value was always with an item within the same proposed domain. The magnitude of the correlation values greater than 0.80 were flagged for possible redundancy and pairs of items with low inter-item correlations (less than 0.3) flagged as potentially not sufficient to warrant inclusion in a summary score.

#### Internal consistency

For both studies, Cronbach’s alpha values for internal consistency reliability were above 0.90 (ranging from 0.91 to 0.96). At baseline of the observational study, values were 0.93 for all HPES-Symptom items and 0.91 for both within-domain subsets of the items, exceeding 0.70 criterion. At Phase 2 trial screening, values were 0.93 for the HPES-Symptom Total Scale and 0.92 for HPES-Physical and 0.96 for HPES-Cognitive domain subsets, thus also exceeding 0.70 criterion. These values provide further support for the hypothesized structure, indicating high internal consistency among the items and evidence for the computation of total and domain-level scores.

#### Summary of scoring

Taken together, the inter-item and item-total correlations, CFA results, and internal consistency coefficients supported the computation of one total and two domain scores for the HPES-Symptom as proposed qualitatively during the instrument development phase. Results based on missing item-level simulations (see Additional file 1) further confirmed the cohesiveness of the item set and provided evidence to support the standard rule of at least 50% item completion to support computation of a summary score. [For the simulations, the mean and SD of each domain score were computed for patients with complete data and then compared to the mean and SD of scores for simulated sets which had a subset of randomly missing items. The scores were considered stable if the 95% CI of the SD value was not outside the range of ±0.10 SD for the complete data.] Furthermore, the developers chose to transform the mean raw scores to a 0-to-100 scale with higher scores indicative of more frequent symptoms. All scale level evaluations used the 0-to-100 scale. The HPES-Symptom Total is not computed if one of the domain scores is missing. The remaining analyses focused on the total and domain-level transformed scores.

#### Test-retest reliability

As shown in Table 2, for participants without treatment changes, ICCs approached the 0.70 criterion for multi-item scales (greater than 0.60 and with 0.70 included within the 95% confidence interval) for the HPES-Symptom Total, Physical and Cognitive scores based on the observational study data [44] and exceeded the 0.70 criterion for all domains in the Phase 2 trial data.

**Table 2:** Observational study HPES-Symptom test-retest reliability, baseline to week 2

HPES-Symptom	ICC (95% CI), n			
	All patients	Participants with no major life change	Participants with no treatment change	Participants with no major life or treatment change
Total	0.64 (0.55–0.72), 185	0.56 (0.45–0.66), 176	0.66 (0.52–0.76), 152	0.60 (0.44–0.72), 146
Physical	0.68 (0.59–0.75), 185	0.65 (0.56–0.73), 176	0.63 (0.51–0.72), 152	0.59 (0.47–0.69), 146
Cognitive	0.56 (0.46–0.65), 185	0.47 (0.34–0.57), 176	0.65 (0.51–0.75), 152	0.60 (0.45–0.71), 146

*CI* confidence interval, *HPES* Hypoparathyroidism Patient Experience Scale, *ICC* intraclass correlation coefficient

#### Construct validity

As shown in Table 3, based on the observational data set, all hypotheses per domain and total score were met with moderate to strong correlations found. Data from the Phase 2 trial confirmed the findings from the observational study.

**Table 3:** Observational study construct validity hypotheses for HPES-Symptom at baseline

HPES-Symptom scale	Supporting measure	Construct validity hypothesis	Correlation values
Total	▪ PGIS-Overall	Moderate to strong correlations with PGIS-Overall scores (to support convergent validity)	0.59
	▪ MFI	Moderate to strong correlations with MFI scores	General Fatigue = 0.63 Physical Fatigue = 0.47 Reduced Motivation = 0.49 Reduced Activity = 0.42 Mental Fatigue = 0.55
Physical	▪ PGIS-Physical ▪ PGIS Cognitive	Moderate to strong correlations with PGIS-Physical scores (to support convergent validity) and less strong correlations with PGIS Cognitive scores (to support divergent validity)	PGIS-Physical = 0.55 PGIS Cognitive = 0.39
	▪ DSQ2-PEM ▪ DSQ2-Sleep disturbances	Moderate to strong correlations with PEM and Sleep-DSQ2 scores	0.72 0.69
Cognitive	▪ PGIS Cognitive ▪ PGIS-Physical	Moderate to strong correlations with PGIS Cognitive scores (to support convergent validity) and less strong correlations with PGIS-Physical scores (to support divergent validity)	PGIS Cognitive = 0.50 PGIS-Physical = 0.43
	▪ CFQ	Moderate to strong correlations with CFQ scores	0.59

*CFQ* Cognitive Failures Questionnaire, *DSQ-2* DePaul Symptoms Questionnaire – Version 2, *HPES* Hypoparathyroidism Patient Experience Scale, *MFI* Multidimensional Fatigue Inventory, *PEM* Post-Exertional Malaise domain (of the DSQ-2), *PGIS* patient global impression of severity

#### Known-groups validity

As shown in Table 4, based on the observational data set, the majority of the hypotheses for the domain and total scores were met. Data from the Phase 2 trial confirmed the findings from the observational study.

**Table 4:** Observational study known-groups validity hypotheses for HPES-Symptom

HPES-symptom domain	Supporting measure	Known-groups validity hypothesis	Statistical evidence				Was the hypothesis met?
			Subgroup	n	LS Means (SE)	ANOVA *P* Value	
Total	PGIS-Overall	Patients with higher levels of severity on PGIS-Overall will have a higher mean score than patients with less severity	1 = No noticeable symptoms/Very mild	18	14.7 (3.54)	Overall < 0.0001 1 vs. 2 0.0035 1 vs. 3 < 0.0001 1 vs. 4 < 0.0001 2 vs. 3 < 0.0001 2 vs. 4 < 0.0001 3 vs. 4 < 0.0001	Yes
			2 = Mild	92	28.1 (1.57)		
			3 = Moderate	160	38.8 (1.19)		
			4 = Severe/Very severe	30	57.1 (2.74)		
Total	SF-36 general health	Patients who report better scores on the SF-36 general health item will have a lower mean score than patients who report worse SF-36 general health scores	1 = Excellent/Very good	62	23.2 (1.96)	Overall < 0.0001 1 vs. 2 < 0.0001 1 vs. 3 < 0.0001 1 vs. 4 < 0.0001 2 vs. 3 < 0.0001 2 vs. 4 < 0.0001 3 vs. 4 0.1606	Yes
			2 = Good	156	34.2 (1.24)		
			3 = Fair	72	47.2 (1.82)		
			4 = Poor	10	58.6 (4.88)		
Total	SDS Days Lost (for RU employment = Yes)	Employed patients with fewer days lost from work (SDS) will have a lower mean score than patients with a greater number of days lost	1 = 25th percentiles or less	84	34.0 (1.70)	0.2534	No
			2 = 75th percentiles or more	72	31.1 (1.84)		
Total	SDS Unproductive Days (for RU employment = Yes)	Employed patients with fewer unproductive days (SDS) will have a lower mean score than patients with a greater number of unproductive days	1 = 25th percentiles or less	120	27.7 (1.43)	< 0.0001	Yes (Not planned within the psychometric analysis plan)
			2 = 75th percentiles or more	77	42.8 (1.79)		
Total	SII Overall	Patients who report greater QOL interference due to their HP overall symptoms will have a higher mean score than patients who report less QOL interference	1 = Not at all/A little	71	21.8 (1.54)	Overall < 0.0001 1 vs. 2 < 0.0001 1 vs. 3 < 0.0001 1 vs. 4 < 0.0001 2 vs. 3 < 0.0001 2 vs. 4 < 0.0001 3 vs. 4 0.1130	Yes
			2 = Some	160	33.3 (1.03)		
			3 = A lot	42	53.4 (2.01)		
			4 = Extremely	27	60.9 (2.51)		
Total	Calcium levels (Question 14)	Patients with lower than normal calcium levels will have higher mean score than patients with normal calcium levels	1 = Much lower than normal	8	58.0 (6.20)	Overall 0.0037 1 vs. 2 0.0016 1 vs. 3 0.0055 1 vs. 4 0.1925 2 vs. 3 0.9655 2 vs. 4 0.9999 3 vs. 4 1.0000	Yes
			2 = Slightly lower than normal	193	34.7 (1.26)		
			3 = Normal	94	36.4 (1.81)		
			4 = Higher than normal	5	36.9 (7.84)		
Physical	PGIS-Physical	Patients with higher levels of severity on PGIS-Physical will have a higher mean score than patients with less severity	1 = No noticeable symptoms/Very mild	20	20.0 (3.63)	Overall < 0.0001 1 vs. 2 0.4848 1 vs. 3 < 0.0001 1 vs. 4 < 0.0001 2 vs. 3 < 0.0001 2 vs. 4 < 0.0001 3 vs. 4 < 0.0001	Yes
			2 = Mild	99	26.5 (1.63)		
			3 = Moderate	140	38.6 (1.37)		
			4 = Severe/Very severe	41	52.9 (2.53)		
Physical	SII Physical	Patients who have greater QOL interference due to their HP physical symptoms will have a higher mean score than patients with less QOL interference	1 = Not at all/A little	144	25.5 (1.12)	Overall < 0.0001 1 vs. 2 0.0002 1 vs. 3 < 0.0001 1 vs. 4 < 0.0001 2 vs. 3 < 0.0001 2 vs. 4 < 0.0001 3 vs. 4 0.0135	Yes
			2 = Some	85	33.2 (1.45)		
			3 = A lot	42	53.6 (2.07)		
			4 = Extremely	29	63.6 (2.49)		
Physical	Calcium levels (Question 14)	Patients with lower than normal calcium levels will have a higher mean score than patients with normal calcium levels (based on Question 14)	1 = Much lower than normal	8	58.6 (6.48)	Overall 0.0026 1 vs. 2 0.0013 1 vs. 3 0.0061 1 vs. 4 0.3043 2 vs. 3 0.8846 2 vs. 4 0.9920 3 vs. 4 0.9998	Yes
			2 = Slightly lower than normal	193	33.8 (1.32)		
			3 = Normal	94	36.2 (1.89)		
			4 = Higher than normal	5	38.7 (8.20)		
Cognitive	PGIS Cognitive	Patients with higher levels of severity on PGIS Cognitive will have a higher mean score than patients with less severity	1 = No noticeable symptoms/Very mild	33	19.1 (3.35)	Overall < 0.0001 1 vs. 2 0.0277 1 vs. 3 < 0.0001 1 vs. 4 < 0.0001 2 vs. 3 < 0.0001 2 vs. 4 < 0.0001 3 vs. 4 0.0823	Yes
			2 = Mild	113	30.0 (1.81)		
			3 = Moderate	107	42.4 (1.86)		
			4 = Severe/Very severe	47	50.7 (2.81)		
Cognitive	SII Cognitive	Patients who report greater impact on QOL interference due to their HP cognitive symptoms will have a higher mean score than patients with less QOL interference	1 = Not at all/A little	117	27.1 (1.61)	Overall < 0.0001 1 vs. 2 0.0108 1 vs. 3 < 0.0001 1 vs. 4 < 0.0001 2 vs. 3 < 0.0001 2 vs. 4 < 0.0001 3 vs. 4 0.3188	Yes
			2 = Some	128	34.1 (1.54)		
			3 = A lot	43	59.8 (2.65)		
			4 = Extremely	12	70.4 (5.03)		

*ANOVA* analysis of variance, *HP* hypoparathyroidism, *HPES* Hypoparathyroidism Patient Experience Scale, *PGIS* patient global impression of severity, *QOL* quality of life, *RU* resource utilization, *SDS* Sheehan Disability Scale, *SE* standard error, *SF 36v2* 36-item Short Form Health Survey, Version 2, *SII* Symptom Impact Items

#### Ability to detect change

Using Phase 2 trial data (see detailed results tables in the Additional file 2), overall, the results were favorable based on a-priori hypotheses indicating the measure is responsive to change:

- Subjects who reported improvement on the PGIS items (overall, physical, and cognitive) showed greater improvement in HPES-Symptom scores than subjects who reported no change or worsening (*P* < 0.05).
- Subjects who reported improvement on the HP-Interference items showed greater improvement in HPES-Symptom scores than subjects who reported no change or worsening *(P <* 0.05).
- As an exploratory hypothesis, subjects who continued taking TransCon PTH and who no longer required SOC showed greater improvement in HPES-Symptom scores than subjects who were still on SOC (*P* < 0.05).

Although not stated as an a-priori hypothesis, results from the CGIS comparisons provided further evidence in support of the HPES-Symptom scores as follows: 1) Subjects who improved based on the CGIS-Cognitive item achieved greater improvements on all three HPES-Symptom scores *(P < 0.05)*; 2) Subjects who improved based on the CGIS-Overall item achieved statistically greater improvement on the HPES-Symptom Total and HPES-Symptom Physical mean scores *(P < 0.05)*; and 3) Subjects who improved based on the CGIS-Physical item achieved statistically greater improvement on the HPES-Symptom Physical mean scores *(P < 0.05)*.

Responsiveness of the HPES-Symptom scores (domains and total) was further assessed by a review of the correlations between the HPES change scores with changes in the PGIS items, CGIS items, the five HP-Interference items, serum and urine calcium levels, and SOC. Overall, the correlation values support the responsiveness of the HPES-Symptom scores. As expected, the correlation values were moderate to strong between the three HPES-Symptom change scores and change in the three PGIS items as well as between the SOC outcome. Correlation values were even larger than expected between the three HPES-Symptom change scores and changes in the five HP-Interference items. However, for the serum and urine calcium levels, the correlation values were in the anticipated direction but trivial to small in magnitude. Additionally, correlation values were consistently moderate to strong for the HPES-Symptom Total and HPES-Symptom Physical change scores with all three CGIS items and moderate between HPES-Symptom Cognitive change and the CGIS-Cognitive item.

#### Threshold for meaningful within-patient change (responder definition)

Table 5 provides the meaningful within-patient change improvement threshold estimates across the methods applied (see the Additional file 2 for additional details). For HPES-Symptom Total, the mean estimate based on a 1-point improvement in the primary anchor, the PGIS-Overall, was approximately 17 points, and similar to the 19 points estimate based on the CGIS Cognitive, the 17 points based on the HP Interference-Quality of life, and the 16 points based on the SOC estimates. The lower-bound distribution-based estimates ranged from 10 to 11 points. For HPES-Symptom Physical, the responder estimate (mean) based on a 1-point improvement in the PGIS-Physical was approximately 17 points and similar to the 19 points based on the PGIS-Overall, the 14 points based on CGIS Overall, the 16 points based on CGIS Physical, the 15 points based on the HP Interference-Quality of life, and the 16 points based on the SOC estimates. The lower-bound distribution-based estimates ranged from 10 to 11 points. Finally, for HPES-Symptom Cognitive, the responder threshold (mean) based on a 1-point improvement in the PGIS Cognitive was approximately 13 points and lower than the 21 points based on the CGIS Cognitive, the 21 points based on the HP Interference-Quality of life, and the 17 points based on the SOC estimates. The lower-bound distribution-based estimates ranged from 15 to 16 points. Given the 15 to 16 range for the distribution-based estimates, the 13-point estimate should be considered with caution. The distribution-based estimates provide an indication of measurement error and, therefore, a responder threshold should be at least larger than this range.

**Table 5:** Interpretation of change – HPES-Symptom

HPES-Symptom			
Meaningful within-patient change	Total	Physical	Cognitive
**Proposed threshold range**	15–17	17–19	15–17
**Anchor-based**			
Primary anchor estimate mean (median)	17 (15)	19 (17)	13 (10)
Supportive anchor estimate minimum (median)	16 (16)	14 (14)	17 (15)
Supportive anchor estimate maximum (median)	19 (20)	17 (17)	21 (25)
**Distribution-based**			
Half-SD	11	11	15
SEM	10	10	16

*HPES* Hypoparathyroidism Patient Experience Scale, *SD* standard deviation, *SEM* standard error measurement

### HPES-Impact measure

#### Descriptive item measurement characteristics and consideration of item reduction

For the observational study, 300 participants completed the HPES-Impact at baseline, and a test-retest sample of 185 completed the measure again approximately 2 weeks after baseline, and for the Phase 2 trial, 59 subjects completed the measure at screening, visit 1 (week 0), and visit 3 (week 4). For both studies, the full 0–4 range (Not at all to Extremely) of item response categories was endorsed for majority of responses by the sample, although several of “Extremely” response categories were not endorsed in Phase 2.

For the observational study, an examination of the item-level response distributions of HPES-Impact items showed no evidence of problematic ceiling effects. For the Phase 2 study, an examination of the response distributions of HPES-Impact items showed some evidence of ceiling effects providing evidence that the impact of HP on the sample tended to be mild. However, for both studies there was no evidence of floor effects.

Correlations greater than 0.80 existed between Item *Moving your body* and Item *Walking*, *r* = 0.81, followed by the correlation of 0.80 between Items *Exercising or doing strenuous activities* and *Physically recovering after doing activities* and also between Items *Tasks around the home* and *Hobbies or leisure activities*. Evaluation of these items using the Phase 2 trial data showed that items remained highly correlated. Given these results, the study team reviewed the qualitative development data for these item pairs. Although participants sometimes experienced these concepts together, the qualitative data provided evidence that these concepts were considered distinct by the participants. Therefore, the decision was made to retain all items.

#### Factor analyses

One 4-factor CFA was conducted using the baseline responses to all HPES-Impact items (Table 6) using the observational study data only. The model fit was acceptable, with RMSEA = 0.078 < 0.10, CFI = 0.960 > 0.95, TLI = 0.956 > 0.95, SRMR = 0.048 < 0.08. All standardized loadings were strong in size, the inter-factor correlations were above 0.80 between the Physical Functioning domain and the Daily Life domain, and between the Daily Life domain and the Social Life and Relationships domain. The remaining inter-factor correlations were greater than 0.70.

**Table 6:** CFA four-factor model—factor loadings (SEs) and fit indices for HPES-Impact using observational study data

Impact Domain/Item	CFA 4-Factor Model Standardized Estimates			
	Factor 1	Factor 2	Factor 3	Factor 4
**Physical Functioning**		**r**_**12**_ **= 0.920**	**r**_**13**_ **= 0.749**	**r**_**14**_ **= 0.799**
1a. Moving your body	0.780* (0.029)	–	–	–
1b. Walking	0.839* (0.023)	–	–	–
1c. Being physically active during the day	0.873* (0.018)	–	–	–
1d. Exercising or doing strenuous activities	0.844* (0.022)	–	–	–
1e. Physically recovering after doing activities	0.877* (0.021)	–	–	–
**Daily Life**			**R**_**23**_ **= 0.780**	**r**_**24**_ **= 0.881**
2a. Tasks around the home	–	0.895* (0.014)	–	–
2b. Hobbies or leisure activities	–	0.820* (0.020)	–	–
2c. Errands	–	0.823* (0.021)	–	–
2d. Complex tasks	–	0.761* (0.027)	–	–
2e. Work or school	–	0.785* (0.031)	–	–
3a. Plan your day around your symptoms	–	0.823* (0.021)	–	–
3b. Take breaks or pace yourself when doing activities	–	0.821* (0.019)	–	–
3c. Stop what you were doing due to your symptoms	–	0.824* (0.021)	–	–
**Psychological Well-Being**				**R**_**34**_ **= 0.794**
4a. Anxious	–	–	0.779* (0.031)	–
4b. Frustrated	–	–	0.869* (0.021)	–
4c. Low self-confidence	–	–	0.726* (0.030)	–
4d. Depressed	–	–	0.769* (0.029)	–
4e. Isolated	–	–	0.756* (0.030)	–
4f. Irritable	–	–	0.796* (0.026)	–
4g. Worried	–	–	0.811* (0.023)	–
4h. Angry	–	–	0.767* (0.026)	–
**Social Life and Relationships**				
5a. Participating in social activities	–	–	–	0.914* (0.014)
5b. The types of activities you do with other people	–	–	–	0.845* (0.019)
5c. Your relationships with friends	–	–	–	0.797* (0.022)
5d. Your relationships with family	–	–	–	0.777* (0.024)
5e. Your relationship with spouse/partner	–	–	–	0.853* (0.027)

*P < 0.05 for H0: loading = 0

CFA = confirmatory factor analysis; CFI = comparative fit index; HPES = Hypoparathyroidism Patient Experience Scale; RMSEA = root mean square error of approximation; SE = standard error; SRMR = standardized root mean residual; TLI = Tucker-Lewis index

Note: *P* value of Chi-square test < 0.0001, RMSEA = 0.078, CFI = 0.960, TLI = 0.956, SRMR = 0.048

Correlations among the items of the HPES-Impact at baseline of the observational study found that the strongest correlation of every item always occurred within the proposed domain, with the magnitude of the correlation values greater than 0.50, indicative of strong relationships and providing further support for the proposed subscales.

#### Internal consistency

For both studies, Cronbach’s alpha values for internal consistency reliability were above 0.70 (ranging from 0.87 to 0.97). In the observational study sample at baseline, values were 0.96 based on all HPES-Impact items and at least 0.87 for the four with-domain subsets of the items (0.87 for Social Life and Relationships, 0.89 for Physical, 0.90 for Daily Life and Psychological Well-Being), exceeding 0.70 criterion. Values at Phase 2 study screening were 0.92 to 0.97 for the HPES-Impact scores.

#### Summary of scoring

Taken together, the inter-item and item-total correlations, CFA results, and internal consistency coefficients supported the computation of one total and four domain scores for the HPES-Impact as proposed qualitatively during the instrument development phase. The standard rule of at least 50% item completion to support computation of a summary score was confirmed by results of missing item-level simulations (see Additional file 1). [For the simulations, the mean and SD of each domain score were computed for patients with complete data and then compared to the mean and SD of scores for simulated sets which had a subset of randomly missing items. The scores were considered stable if the 95% CI of the SD value was not outside the range of ±0.10 SD for the complete data.] All scale level evaluations used 0-to-100 scaled total and domain-level scores based on a transformation of the mean raw scores. The HPES-Impact Total is not computed if any of the domain scores are missing. Higher scores are indicative of greater impact.

#### Test-retest reliability

For both studies, ICCs were either greater than 0.70 criterion or 0.70 was included within the 95% confidence interval for all domains across all subjects without major life events or treatment changes except for the Physical Functioning domain (Table 7).

**Table 7:** Observational study HPES-Impact test-retest reliability, baseline to week 2

HPES-Impact	ICC (95% CI), n			
	All patients	Patients with no major life change	Patients with no treatment change	Patients with no major life or treatment change
Total	0.70 (0.61–0.77), 185	0.68 (0.58–0.75), 176	0.74 (0.61–0.83), 152	0.72 (0.57–0.82), 146
Physical functioning	0.49 (0.37–0.59), 185	0.45 (0.32–0.56), 176	0.52 (0.39–0.63), 152	0.46 (0.32–0.58), 146
Daily life	0.70 (0.60–0.77), 185	0.68 (0.57–0.76), 176	0.73 (0.59–0.82), 152	0.71 (0.54–0.81), 146
Psychological well-being	0.60 (0.48–0.70), 185	0.59 (0.47–0.69), 176	0.61 (0.38–0.75), 152	0.61 (0.37–0.75), 146
Social life and relationships	0.66 (0.57–0.73), 185	0.64 (0.54–0.72), 176	0.70 (0.57–0.79), 152	0.70 (0.54–0.80), 146

*CI* confidence interval, *HPES* Hypoparathyroidism Patient Experience Scale, *ICC* intraclass correlation coefficient

#### Construct validity

As shown in Table 8, based on the observational data set, the majority of the hypotheses were met with moderate to strong correlations found. Data from the Phase 2 trial confirmed the findings from the observational study.

**Table 8:** Observational study construct validity hypotheses for HPES-Impact at baseline

HPES-Impact measure	Supporting measure	Construct validity hypothesis	Correlations
Total	▪ SII	Moderate to strong correlation with interference with QOL due to HP symptoms (SII)	SII Overall = 0.76
Physical functioning	▪ SF-36 PCS ▪ SF-36 MCS	Moderate to strong correlations with SF-36 PCS scores (to support convergent validity) and less strong correlations with SF-36 MCS scores (to support divergent validity)	PCS = − 0.75 MCS = − 0.34
	▪ HP-interference	Moderate to strong correlations with HP-Interference physical functioning scores	Physical functioning = 0.71
Daily life	▪ HP-interference	Moderate to strong correlations with SDS scores	SDS = 0.77
		Moderate to strong correlations with HP-Interference daily life scores	Daily functioning = 0.77
Psychological well-being	▪ HADS	Moderate to strong correlations with HADS anxiety and depression scores	Depression = 0.46 Anxiety = 0.54
	▪ SF-36 PCS ▪ SF-36 MCS	Moderate to strong correlations with SF-36 MCS scores (to support convergent validity) and less strong correlations with SF-36 PCS scores (to support divergent validity)	PCS = −0.46 MCS = − 0.62
	▪ HP-interference	Moderate to strong correlations with HP-Interference emotional scores	Emotional Well-Being = 0.66
Social life and relationships	▪ MOS-SSS	Moderate to strong correlations with MOS-SSS domain scores	Emotional/informational = −0.22 Tangible = − 0.26 Affectionate = − 0.12 Positive social interaction = −0.21 Total = − 0.24
	▪ Friends and family support	Moderate to strong correlations between with the family support item scores	−0.23
	▪ HP-interference	Moderate to strong correlations with HP-Interference social functioning scores	Social functioning = 0.76

*HADS* Hospital Anxiety and Depression Scale, *HP* hypoparathyroidism; *HPES* Hypoparathyroidism Patient Experience Scale, *MCS* Mental component summary, *MOS-SSS* Medical Outcome Study-Social Support Survey, *PCS* Physical component summary, *QOL* quality of life, *SDS* Sheehan Disability Scale, *SF-36v2* 36-item Short Form Health Survey, Version 2, *SII* Symptom Impact Items

#### Known-groups validity

As shown in Table 9, based on the observational data set, results provided strong evidence for known-groups validity, with at least one hypothesis per domain and total score were met. Data from the Phase 2 trial generally confirmed the findings from the observational study.

**Table 9:** Observational study known-groups validity hypotheses for HPES-Impact

HPES-impact	Supporting measure	Known-groups validity hypothesis	Statistical evidence				Was the hypothesis met?
			Subgroup	n	LS Means (SE)	ANOVA *P* value	
Total	PGIS-Overall	Patients with higher levels of severity on PGIS-Overall will have a higher mean score than patients with less severity	1 = No noticeable symptoms/Very mild	18	7.8 (3.25)	Overall < 0.0001 1 vs. 2 0.0007 1 vs. 3 < 0.0001 1 vs. 4 < 0.0001 2 vs. 3 < 0.0001 2 vs. 4 < 0.0001 3 vs. 4 < 0.0001	Yes
			2 = Mild	92	21.6 (1.44)		
			3 = Moderate	160	38.3 (1.09)		
			4 = Severe/Very severe	30	56.1 (2.52)		
	PGIS-Physical	Patients with higher levels of severity on the PGIS-Physical will have a higher mean score than patients with less severity	1 = No noticeable symptoms/Very mild	20	7.1 (3.20)	Overall < 0.0001 1 vs. 2 < 0.0001 1 vs. 3 < 0.0001 1 vs. 4 < 0.0001 2 vs. 3 < 0.0001 2 vs. 4 < 0.0001 3 vs. 4 < 0.0001	Yes
			2 = Mild	99	24.3 (1.44)		
			3 = Moderate	140	37.4 (1.21)		
			4 = Severe/Very severe	41	52.8 (2.24)		
	PGIS Cognitive	Patients with higher levels of severity on the PGIS Cognitive will have a higher mean score than patients with less severity	1 = No noticeable symptoms/Very mild	33	13.4 (2.72)	Overall < 0.0001 1 vs. 2 < 0.0001 1 vs. 3 < 0.0001 1 vs. 4 < 0.0001 2 vs. 3 < 0.0001 2 vs. 4 < 0.0001 3 vs. 4 0.0098	Yes
			2 = Mild	113	28.1 (1.47)		
			3 = Moderate	107	38.4 (1.51)		
			4 = Severe/Very severe	47	47.1 (2.28)		
Physical Functioning	Physically active (Question 18)	Patients who report being less physically active will have a higher mean score than patients who are more physically active	1 = Not at all	9	57.8 (6.61)	Overall < 0.0001 1 vs. 2 0.0776 1 vs. 3 0.0018 1 vs. 4 < 0.0001 2 vs. 3 0.0457 2 vs. 4 < 0.0001 3 vs. 4 0.0311	Yes
			2 = A little	73	40.3 (2.32)		
			3 = Some	174	32.9 (1.50)		
			4 = A lot/Extremely	44	23.5 (2.99)		
Daily Life	Energy (Question 19)	Patients with lower energy (greater impact) levels during the day will have a higher mean score than patients with higher energy levels	1 = Not at all	13	69.6 (5.07)	Overall < 0.0001 1 vs. 2 < 0.0001 1 vs. 3 < 0.0001 1 vs. 4 < 0.0001 2 vs. 3 0.0062 2 vs. 4 < 0.0001 3 vs. 4 0.1217	Yes
			2 = A little	136	37.8 (1.57)		
			3 = Some	121	30.2 (1.66)		
			4 = A lot/Extremely	30	21.6 (3.34)		
	Employment status	Patients who report being currently employed will have a higher mean score than patients who are unemployed	1 = Yes	250	30.9 (1.18)	Overall < 0.0001 1 vs. 2 < 0.0001	No; but the hypothesis was flawed. The results are logical (less impact may lead to being able to work)
			2 = No	50	52.3 (2.65)		
Social Life and Relationships	Friends and Family (Question 20)	Patients who report less family support will have a higher mean score than patients with more family support	1 = Not at all	12	48.3 (6.06)	Overall 0.0022 1 vs. 2 0.2912 1 vs. 3 0.1162 1 vs. 4 0.0109 1 vs. 5 0.0050 2 vs. 3 0.9996 2 vs. 4 0.2999 2 vs. 5 0.1418 3 vs. 4 0.4792 3 vs. 5 0.2350 4 vs. 5 0.9982	Yes
			2 = A little	70	34.4 (2.51)		
			3 = Some	155	32.5 (1.69)		
			4 = A lot	42	25.7 (3.24)		
			5 = Extremely	21	21.6 (4.58)		
Psychological Well-Being	Number of comorbid issues (Question 15)	Patients who report a higher number of comorbidity issues will have a higher mean score than patients with fewer comorbidity issues	1 = 25th percentile or less	174	31.7 (1.42)	1 vs. 2 0.0026	Yes
			2 = 75th percentile or more	81	39.4 (2.08)		

*ANOVA* analysis of variance, *HPES* Hypoparathyroidism Patient Experience Scale, *LS* least squares, *PGIS* patient global impression of severity

#### Ability to detect change

Patterns of mean changes in the HPES-Impact scores using the Phase 2 trial data were compared for groups based on changes in the three PGIS and three CGIS items (overall, physical, and cognitive), changes in the five HP-Interference items, improvement in serum and urine calcium levels (normal), and SOC (on/off) (see detailed results tables in the Additional file 2). Overall, the hypotheses were met:

- Subjects who reported improvement on the PGIS items (overall, physical, and cognitive) showed greater improvement in HPES-Impact scores than subjects who reported no change or worsening. This hypothesis was supported for the HPES-Impact Total, HPES-Physical Functioning, HPES-Impact Daily Life, and HPES-Impact Social Life and Relationships scores for all three PGIS items and for HPES-Impact Psychological Well-Being scores for PGIS Cognitive item (*P* < 0.05)*.*
- Subjects who reported improvement on the HP-Interference items showed greater improvement in HPES-Impact scores than subjects who reported no change or worsening (*P* < 0.05).

Although not stated as an a-priori hypothesis, results from the CGIS comparisons provided further evidence in support of the HPES-Impact scores as follows:

- Subjects who had improved based on clinician-reported change on the CGIS-Cognitive item achieved greater improvements on all five HPES-Impact scores (*P* < 0.05).
- None of the comparisons were statistically significant for the CGIS-Overall and CGIS-Physical items although the pattern in the means was in the anticipated direction.

The exploratory hypothesis that subjects who continued taking TransCon PTH and who no longer required SOC, compared to subjects who remained on SOC, would show greater improvement was not supported although the direction of the mean change scores was in the anticipated direction.

Responsiveness of the HPES-Impact scores (domains and total) was further assessed by a review of the correlations between the HPES change scores with changes in the PGIS items, CGIS items, the five HP-Interference items, serum and urine calcium levels, and SOC. Overall, the correlation values support the responsiveness of the HPES-Impact scores. As expected, the correlation values were at least moderate for the five HPES-Impact scores and the three PGIS items. Correlation values were even larger between the HPES-Impact scores and the five HP-Interference items. The correlation was moderate between the HPES-Impact scores and the SOC outcome. However, for the serum and urine calcium levels, the correlation values were in the anticipated direction but trivial to small in magnitude. For the CGIS items, results were consistently moderate for the HPES-Impact Total and HPES-Impact Social Life and Relationships and small to strong for the remaining scales.

#### Threshold for meaningful within-patient change (responder definition)

A review of the meaningful within-patient change improvement (across methods and between the mean and median values) provides a range of thresholds (see the Additional file 2 for additional details). The following key results were observed:

- For HPES-Impact Total, the mean estimate based on a 1-point improvement in the primary anchor, the HP-Interference Quality of Life, was approximately 13 points, and similar to the 16 points estimate based on the PGIS-Overall, 15 points based on the CGIS Cognitive, and 13 points based on the SOC estimate. The lower-bound distribution-based estimates were both approximately 11 points.
- For HPES-Impact Physical Function, the responder estimate (mean) based on a 1-point improvement in the primary anchor, the HP-Interference Physical Functioning, was approximately 13 points and aligned to the 13 points based on HP Interference Quality of Life, lower than the 18 points based on PGIS-Overall and higher than the estimates based on PGIS-Physical Symptoms (12 points) and CGIS Physical Symptoms (11 points), similar to the estimates based on SOC (14 points) and slightly lower than the distribution-based estimates, which ranged from 13 to 14 points.
- The primary threshold estimate for HPES-Impact Daily Life was 14 points based on HP-Interference Daily Functioning with supporting anchor-based estimates ranging from 12 points based on PGIS Cognitive to 17 points based on CGIS Cognitive. The distribution-based estimates were approximately 13 points.
- HPES-Impact Psychological Well-Being’s primary estimate was based on HP-Interference Emotional Well-Being and was 16 points. This estimate was supported by values from 4 points based on PGIS Cognitive Symptoms to 17 points based on CGIS Cognitive Symptoms and distribution-based estimates ranging from 11 to 15 points; and finally, for HPES-Impact Social Life and Relationships, the responder threshold (mean) based on a 1-point improvement in the primary anchor, the HP Interference Social Functioning item, was approximately 8 points, which aligned with the 8 points based on the PGIS Cognitive Symptoms item but was lower than the remaining estimates, which ranged from 10 points based on SOC to 12 based on the HP Interference-Quality of Life. Given the 11- to 14-point range for the distribution-based estimates, the 8-point estimate for the HPES-Impact Social Life and Relationship domain should be considered with caution. The distribution-based estimates provide an indication of measurement error and, therefore, a responder threshold should be at least larger than this range.

### HPES exploratory results from the TCP-201 PaTH Forward phase 2 clinical trial

Symptom total score and domain scores demonstrated statistically significant improvements (i.e., decrease in scores) for TransCon PTH compared to placebo (Table 10). Additionally, from baseline to week 4, the difference in mean HPES-Impact total score and domain scores demonstrated statistically significant improvements (i.e., a decrease in score) for TransCon PTH compared to placebo (Table 10).

**Table 10:** Summary of HPES-Impact and HPES-Symptom Scales for TransCon PTH vs. placebo by ANCOVA (Full analysis set; *N* = 59)

HPES	TransCon PTH All Subjects (***n*** = 44)	Placebo (***n*** = 15)
**HPES-Impact **		
Daily life domain, n	44	15
Baseline, mean (SE)	29.6 (3.9)	36.8 (7.4)
Week 4, mean (SE)	13.5 (2.5)	35.7(9.9)
LS Mean (SE)	−16.8 (2.7)	0.8 (4.6)
Difference in LS Mean (SE)	−17.7 (5.3)	
*P* value	0.0015	
Physical functioning domain (n)	44	15
Baseline, mean (SE)	32.6 (3.9)	41.3 (7.6)
Week 4, mean (SE)	17.7 (3.0)	38.9 (9.3)
LS Mean (SE)	−15.8 (2.9)	0.3 (5.1)
Difference in LS mean (SE)	−16.1 (5.8)	
*P* value	0.0077	
Psychological Well-Being Domain (n)	44	15
Baseline, mean (SE)	24.8 (3.3)	25.0 (6.2)
Week 4, mean (SE)	12.4 (2.3)	25.0 (7.1)
LS Mean (SE)	−12.5 (2.6)	0.1 (4.5)
Difference in LS mean (SE)	−12.6 (5.1)	
*P* value	0.0163	
Social life and relationship domain (n)	44	15
Baseline, mean (SE)	22.0 (3.4)	24.7 (5.6)
Week 4, mean (SE)	10.6 (2.6)	29.0 (9.8)
LS mean (SE)	−11.7 (2.8)	4.9 (4.9)
Difference in LS mean (SE)	−16.6 (5.6)	
*P* value	0.0045	
Total HPES-Impact score (n)	44	15
Baseline, mean (SE)	27.3 (3.3)	32.0 (5.8)
Week 4, mean (SE)	13.5 (2.3)	32.1 (8.7)
LS mean (SE)	−14.2 (2.5)	1.5 (4.3)
Difference in LS mean (SE)	−15.7 (5.0)	
*P* value	0.0026	
**HPES-Symptom **		
Physical Domain (n)	44	15
Baseline, mean (SE)	37.3 (3.2)	43.2 (6.3)
Week 4, mean (SE)	20.3 (2.3)	42.1 (8.4)
LS Mean (SE)	−17.5 (2.3)	0.4 (4.0)
Difference in LS Mean (SE)	−18.1 (4.6)	
*P* value	0.0003	
Cognitive Domain (n)	44	15
Baseline, mean (SE)	38.6 (4.7)	34.7 (7.5)
Week 4, mean (SE)	20.5 (3.0)	39.7 (6.9)
LS Mean (SE)	−17.6 (2.8)	3.2 (4.9)
Difference in LS Mean (SE)	−20.8 (5.6)	
*P* value	0.0005	
Total HPES-Symptom Score (n)	44	15
Baseline, mean (SE)	38.0 (3.5)	38.9 (5.4)
Week 4, mean (SE)	20.4 (2.4)	40.9 (7.2)
LS Mean (SE)	−17.7 (2.3)	2.3 (4.0)
Difference in LS Mean (SE)	−20.0 (4.6)	
*P* value	< 0.0001	

*ANCOVA* analysis of covariance; *HPES* Hypoparathyroidism Patient Experience Scale, *LS* least squares; *SE* Standard error

## Discussion

The data from the observational study provided an opportunity to conduct an initial psychometric evaluation of the HPES measures. The evaluation was planned and implemented in accordance with the recommendations outlined in the FDA PRO guidance [26] and then expanded to include a longitudinal evaluation using data from an ongoing Phase 2 clinical trial.

Within the context of the observational study, a review of the descriptive statistics for the HPES-Symptom provided evidence for adequate item performance with no limiting distributional anomalies or response biases at baseline or at week 2. Furthermore, as expected, the item scores were stable in the 2-week observational period. A review of the structure of the HPES-Symptom focusing on inter-item correlations and CFA results provided support for the proposed structure of a Total score accompanied by Physical and Cognitive domains scores. One item pair was flagged for potential item redundancy (*Feeling tired* and *Low energy*) with a correlation value above 0.80. Evaluation of these items using the Phase 2 study data showed that these two items remained highly correlated (*r* = 0.96 and with similar responsiveness). However, targeted review of participant feedback during the qualitative development of the HPES-Symptom should be considered to identify evidence that participants approach these two concepts in a distinct manner and the decision was made to retain both items.

Overall, for the HPES-Symptom, the Total and domain scores demonstrated acceptable reliability and validity measurement properties for both the observational and the Phase 2 study samples. Internal consistency evidence was strong. Test-retest reliability estimates generally approached the recommended 0.70 threshold. For construct validity, the patterns of correlations with other PRO measures were mainly as hypothesized, thus supporting the HPES-Symptom scores and the constructs measured. Mean HPES-Symptom scores also differed as anticipated and significantly across known-groups based on the SF-36v2 general health score, SII scores, and PGIS scores, thus providing evidence for the scores discriminating between meaningful groups. Results were not as strong but still in the general direction when evaluated using the SDS days lost and calcium levels. Although small in size, the Phase 2 clinical trial data confirmed the cross-sectional and test-retest properties.

Despite the small sample, results from the Phase 2 clinical trial provide some evidence supporting the ability of the HPES-Symptom total and domain scores to detect change. The ANOVA and responsiveness correlation results between the HPES-Symptom change scores and the changes in supporting measures met expectations for most comparisons. Non-significant correlations for the measure and biomarkers which were in the anticipated direction may have been due to the small sample size.

The Phase 2 clinical trial data offered the first opportunity to develop thresholds for meaningful within-patient change for the HPES-Symptom using anchor- and distribution-based methods. Results from these analyses provide evidence for a range of 15 to 19 points as thresholds for characterizing meaningful within-patient improvement on HPES-Symptom total and domain scores (transformed). These estimates are based on a sample of subjects who were receiving SOC treatment and reported additional meaningful benefit in these concepts between baseline and visit 3. In future application, the lower end of the range may be more appropriate for a milder patient population while the higher threshold values may be more appropriate for a more symptomatic patient population.

Regarding the HPES-Impact scale, within the context of the observational study, a review of the descriptive statistics provided evidence for adequate item performance with no limiting distributional anomalies or response biases at baseline or at week 2. Furthermore, as expected, the item scores were reasonably stable in the 2-week observational period. A review of the structure of the HPES-Impact focusing on inter-item correlations and CFA results provided support for the proposed structure of a total score accompanied by additional domain scores. Furthermore, the factors are all highly related with inter-factor correlations ranging from 0.75 to 0.92 which may suggest redundancy in the domain and total scores. The following three item pairs were flagged for potential redundancy: Item *Moving your body* and Item *Walking*; Item *Exercising or doing strenuous activities* and Item *Physically recovering after doing activities*; and Item *Tasks around the home* and Item *Hobbies or leisure activities*. Evaluation of these items using the Phase 2 trial data showed that these items remained highly correlated and shared similar responsiveness. Targeted review of participant feedback during the qualitative development of the HPES-Impact should be considered to identify evidence that subjects approach these two concepts in a distinct manner and the decision was made to retain these items. Overall, the Total and domain scores demonstrated acceptable reliability and validity measurement properties for both the observational and Phase 2 trial samples. Internal consistency evidence was strong. Test-retest reliability estimates generally approached the recommended 0.70 threshold, except for the Physical Functioning Domain. A future study is planned to further evaluate test-retest which will include a more appropriate stability criterion for physical functioning. For construct validity, the patterns of correlations with other PRO measures were mainly as hypothesized, thus supporting the HPES-Impact scores and the constructs measured. Mean HPES-Impact scores also differed as anticipated and significantly across known-groups based on the PGIS scores, physically active and energy questions, employment status, level of family support, and number of comorbid issues. The mean differences for subgroups defined by these external measures provided evidence to support the discriminating ability of the HPES-Impact scores. Although small in size, the Phase 2 trial data confirmed the cross-sectional and test-retest properties.

As with the HPES-Symptom measure, despite the small sample, results from the Phase 2 trial support the HPES-Impact total and domain scores’ ability to detect change. The ANOVA and responsiveness correlation results between the HPES-Impact change scores and the changes in supporting measures met expectations for most comparisons. Non-significant correlations with the measure and SOC which were in the anticipated direction may have been due to the small sample size.

The Phase 2 trial data offered the first opportunity to develop thresholds for meaningful within-patient change for the HPES-Impact measure using anchor- and distribution-based methods. Results from these analyses provide evidence for a range of 13 to 18 points as thresholds for characterizing meaningful within-patient improvement on HPES-Symptom total and domain scores (transformed). In future application, the lower end of the range may be more appropriate for a milder patient population while the higher threshold values may be more appropriate for a more symptomatic patient population.

### Clinical implications of the development of the HPES measures

Several recent studies have demonstrated that patients with HP treated with the conventional therapy (oral calcium and vitamin D supplements) have reduced quality of life (QOL) compared to either suitable controls or general population [1, 7, 8, 12, 21, 50, 51]. These findings indicate that the assessment and improvement in QOL should be a priority for clinicians caring for patients with HP to provide an optimal management of HP. Additionally, European Society of Endocrinology guidelines on treatment of chronic HP in adults recommend personalizing treatment and focus on the overall well-being and QOL improvement of patients with HP to achieve the therapeutic goals to treat HP. According to the guidelines, QOL is one of the critical outcomes to improve in patients with HP [52]. The HPES findings from the phase 2 trial, showing improvement in both symptoms and impacts, provide evidence that appropriate treatment can significantly improve the lives of these patients.

Further, the additional illness burden of impaired daily activities has been one of the major concerns expressed by patients with HP and clinical experts of HP have emphasized that further studies are required to quantify the effect of HP on patients’ QOL. Using disease-specific questionnaires and the HPES disease-specific measures, developed in compliance with FDA PRO guidance, can be instrumental to assess symptoms of HP from patients’ perspectives and impact of treatment from the clinical perspective. With the promising implications, the HPES measures may positively impact the clinical outcome in management of adults with HP.

## Conclusions

In summary, both the HPES-Symptom and HPES-Impact, developed according to FDA PRO guidance, have been found to be conceptually sound with adequate evidence to support reliability and validity of the measures. Phase 2 trial results supported both HPES total and domain scores ability to detect a change. The difference in mean HPES-Symptom and HPES-Impact total and domain scores demonstrated statistically significant improvements for TransCon PTH compared to placebo despite the small sample and a short 4-week duration on fixed, non-optimized doses. Understanding and measuring the impact of treatment, which are important for patients and adequately reflect their experience living with HP, is critical to assessing treatment benefit as well as improving provider-patient communication. Incorporation of the HPES measures into both clinical and research settings will help to further elucidate and assess the patient experience of living with HP.

## Supplementary Information

**Additional file 1: Figure S1.** Missing-Data Simulations: Scatterplot of SDs of Baseline HPES-Symptom and HPES-Impact Total and Domain Scores Group-level Mean of the SD in Simulated (500X) Scores Computed with Different Numbers of Missing Item Responses Among Subjects with Complete Data (*N* = 223 to 300).

**Additional file 2.**

## Data Availability

The data for the research presented in the publication may be available on a case-by-case basis for reasonable requests from the corresponding author.

## Abbreviations

ANCOVA: Analysis of covariance
ANOVA: Analysis of variance
CASR: Calcium-sensing receptor
CFA: Confirmatory factor analysis
CFI: Comparative fit index
CFQ: Cognitive Failures Questionnaire
CGIS: Clinical Global Impression of Severity
DSQ-2: DePaul Symptom Questionnaire - Version 2
FDA: U. S. Food & Drug Administration
HADS: Hospital Anxiety and Depression Scale
HP: Hypoparathyroidism
HPES: Hypoparathyroidism Experience Scale
HPES-Impact: Hypoparathyroidism Experience Scale-Impact
HPES-Symptom: Hypoparathyroidism Experience Scale-Symptom
HRQOL: Health-related quality of life
ICC: Intraclass correlation coefficient
IRB: Institutional Review Board
MFI: Multidimensional Fatigue Inventory
MOS-SSS: MOS Social Support Survey
PGIS: Patient Global impression of Severity
PRO: Patient-reported outcome
PTH: Parathyroid hormone
QOL: Quality of life
RMSEA: Root mean square error of approximation
SD: Standard deviation
SDS: Sheehan Disability Scale
SII: Symptom Impact Items
SOC: Standard of Care
SRMR: Standardized root mean residual
TLI: Tucker-Lewis Index

## Footnotes

1 Based on a *JPRO* reviewer’s comment of this manuscript, future evaluations will include consideration of Coefficient ω (cf. McDonald, 1999, Chapter 6).
