# Supplementary material for: Psychometric validation of the Hypoparathyroidism Patient Experience Scales (HPES)
Source: J Patient Rep Outcomes. 2021 Aug 10;5:70. doi: 10.1186/s41687-021-00320-2 (PMC8355305; doi:10.1186/s41687-021-00320-2)

# Additional file 1

***Fig. S1.*** *Missing-Data Simulations: Scatterplot of SDs of Baseline HPES Symptom and Impact Total and Domain Scores Group-level Mean of the SD in Simulated (500X) Scores Computed with Different Numbers of Missing Item Responses Among Subjects with Complete Data (N = 223 to 300)*


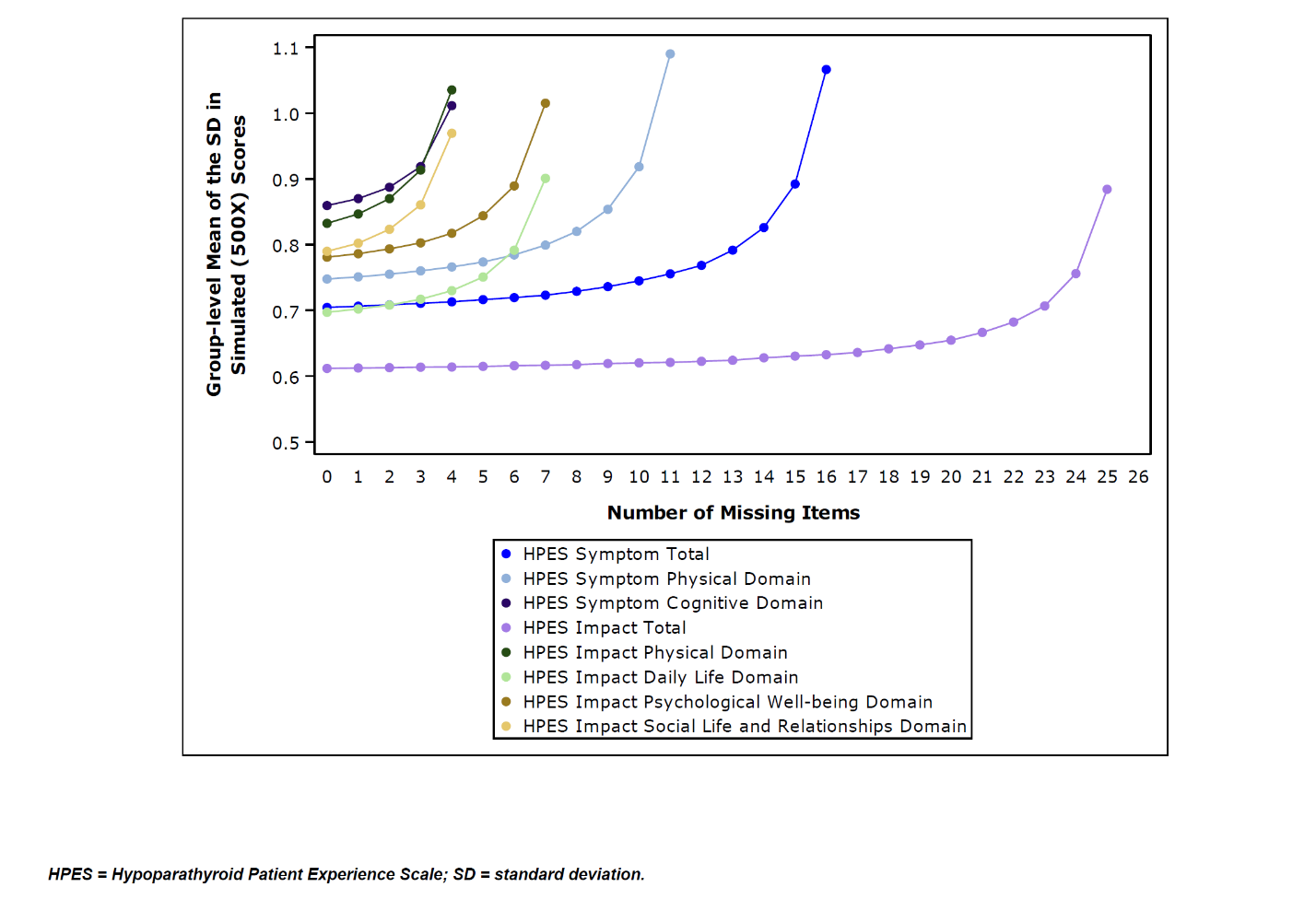

Supplement: Supplementary file 1 — Additional file 1: Figure S1. Missing-Data Simulations: Scatterplot of SDs of Baseline HPES-Symptom and HPES-Impact Total and Domain Scores Group-level Mean of the SD in Simulated (500X) Scores Computed with Different Numbers of Missing Item Responses Among Subjects with Complete Data (N = 223 to 300). [file 41687_2021_320_MOESM1_ESM.docx]
