# Supplementary material for: Psychometric validation of the Hypoparathyroidism Patient Experience Scales (HPES)
Source: J Patient Rep Outcomes. 2021 Aug 10;5:70. doi: 10.1186/s41687-021-00320-2 (PMC8355305; doi:10.1186/s41687-021-00320-2)

Additional file 2

Table of Contents

[Additional file 2 1](#_Toc68702363)

[HPES Symptom - Ability to Detect Change 3](#_Toc68702364)

[**Table S1:** Phase 2 Study HPES-Symptom: Ability to Detect Change by PGIS 3](#_Toc68702365)

[**Table S2:** Phase 2 Study HPES-Symptom: Ability to Detect Change by HP-Interference Items 4](#_Toc68702366)

[HPES Symptom - Threshold for Meaningful Within-Patient Change (Responder Definition) 5](#_Toc68702367)

[**Table S3:** Phase 2 Study Interpretation of Change–HPES-Symptom Total 5](#_Toc68702368)

[**Table S4:** Phase 2 Study Interpretation of Change–HPES-Symptom Physical 7](#_Toc68702369)

[**Table S5.** Phase 2 Study Interpretation of Change – HPES-Symptom Cognitive 9](#_Toc68702370)

[**Fig. S1:** Cumulative Distribution Function of Change in HPES Symptom Total Score from Screening to Visit 3, by change in PGIS-Overall Symptoms 11](#_Toc68702371)

[**Fig. S2:** Probability Density Function of Change in HPES Symptom Total Score from Screening to Visit 3, by Change in PGIS-Overall Symptoms 12](#_Toc68702372)

[**Fig. S3**. Cumulative Distribution Function of Change in HPES Symptom Physical Score from Screening to Visit 3, by Change in PGIS-Physical Symptom 13](#_Toc68702373)

[**Fig. S4.** Probability Density Function of Change in HPES Symptom Physical Score from Screening to Visit 3, by Change in PGIS-Physical Symptoms 14](#_Toc68702374)

[**Fig. S5.** Cumulative Distribution Function of Change in HPES Symptom Cognitive Score from Screening to Visit 3, by Change in PGIS-Cognitive Symptoms 15](#_Toc68702375)

[**Fig. S6.** Probability Density Function of Change in HPES Symptom Cognitive Score from Screening to Visit 3, by Change in PGIS-Cognitive Symptoms 16](#_Toc68702376)

[HPES Impact - Ability to Detect Change 17](#_Toc68702377)

[**Table S6.** Phase 2 Study HPES-Impact: Ability to Detect Change by PGIS 17](#_Toc68702378)

[**Table S7**. Phase 2 Study HPES-Impact: Ability to Detect Change by HP-Interference Items 19](#_Toc68702379)

[HPES Impact - Threshold for Meaningful Within-Patient Change (Responder Definition) 21](#_Toc68702380)

[**Table S8.** Phase 2 Study Interpretation of Change – HPES-Impact Total 21](#_Toc68702381)

[**Table S9.** Phase 2 Study Interpretation of Change–HPES-Impact Physical Function 23](#_Toc68702382)

[**Table S10.** Phase 2 Study Interpretation of Change – HPES-Impact Daily Life 25](#_Toc68702383)

[**Table S11.** Phase 2 Study Interpretation of Change – HPES-Impact Psychological Well-Being 27](#_Toc68702384)

[**Table S12.** Phase 2 Study Interpretation of Change – HPES-Impact Social Life and Relationships 29](#_Toc68702385)

[**Fig. S7.** Cumulative Distribution Function of Change in HPES Impact Total Score from Screening to Visit 3, by HP interference-Quality of Life 31](#_Toc68702386)

[**Fig. S8.** Probability Density Function of Change in HPES Impact Total Score from Screening to Visit 3, by HP interference- Quality of Life 32](#_Toc68702387)

[**Fig. S9.** Cumulative Distribution Function of Change in HPES Impact Physical Functioning Score from Screening to Visit 3, by HP interference-Physical functioning 33](#_Toc68702388)

[**Fig. S10.** Probability Density Function of Change in HPES Impact Physical Functioning Score from Screening to visit 3, by HP interference-Physical functioning 34](#_Toc68702389)

[**Fig. S11.** Cumulative Distribution Function of Change in HPES Impact Daily Life Score from Screening to Visit 3, by HP interference–Daily Functioning 35](#_Toc68702390)

[**Fig. S12.** Probability Density Function of Change in HPES Impact Daily Life Score from Screening to Visit 3, HP Interference–Daily Functioning 36](#_Toc68702391)

[**Fig. S13.** Cumulative Distribution Function of Change in HPES Impact Psychological Well-Being Score from Screening to Visit 3, by HP interference-Emotional Well-being 37](#_Toc68702392)

[**Fig. S14.** Probability Density Function of Change in HPES Impact Psychological Well-Being Score from Screening to Visit 3, by HP interference-Emotional Well-being 38](#_Toc68702393)

[**Fig. S15.** Cumulative Distribution Function of Change in HPES Impact Social Life and Relationships Score from Screening to Visit 3, by HP interference-Social Functioning 39](#_Toc68702394)

[**Fig. S16.** Probability Density Function of Change in HPES Impact Social Life and Relationships Score from Screening to Visit 3, by HP interference-Social Functioning 40](#_Toc68702395)

HPES Symptom - Ability to Detect Change

### **Table S1:** Phase 2 Study HPES-Symptom: Ability to Detect Change by PGIS

| **Score/Group** | **Improved** | | **No Change/Worsening** | |  | | |
| --- | --- | --- | --- | --- | --- | --- | --- |
|  | **n** | **LS Mean (SE), Median, Min, Max, Lower 95% CI, Upper 95% CI** | **n** | **LS Mean (SE), Median, Min, Max, Lower 95% CI, Upper 95% CI** | **Cohen’s *d*** | **t-statistic (*P* Value)** | **ANOVA F-Statistic (*P* Value)** |
| **HPES-Symptom Total (0-100 scale)** |  |  |  |  |  |  |  |
| PGIS-Overall Symptoms | 29 | −21.39 (3.4), −17.71, −68.13, 11.25, −28.15, −14.64 | 27 | −3.31 (3.5), −2.08, −38.33, 39.17, −10.76, 4.14 | −0.96 | −3.70 (0.0005) | 13.69 (0.0005) |
| PGIS-Physical Symptoms | 28 | −20.44 (3.6), −15.63, −68.13, 11.25, −27.48, −13.40 | 28 | −4.91 (3.6), −3.13, −40.83, 39.17, −12.50, 2.68 | −0.79 | −3.08 (0.0033) | 9.48 (0.0033) |
| PGIS Cognitive Symptoms | 28 | −21.62 (3.5), −18.75, −68.13, 19.58, −29.17, −14.07 | 28 | −3.73 (3.5), −3.54, −38.33, 39.17, −10.36, 2.91 | −1.05 | −3.65 (0.0006) | 13.35 (0.0006) |
| **HPES-Symptom Physical Domain (0-100 scale)** |  |  |  |  |  |  |  |
| PGIS-Overall Symptoms | 29 | −23.13 (2.9), −18.75, −56.25, −4.17, −28.42, −17.84 | 27 | −2.55 (3.0), −2.08, −41.67, 29.17, −9.25, 4.15 | −1.22 | −4.98 (< 0.0001) | 24.84 (< 0.0001) |
| PGIS-Physical Symptoms | 28 | −21.95 (3.1), −19.79, −56.25, 0.00, −27.66, −16.24 | 28 | −4.46 (3.1), −6.25, −41.67, 29.17, −11.41, 2.48 | −0.98 | −3.99 (0.0002) | 15.91 (0.0002) |
| PGIS Cognitive Symptoms | 28 | −18.60 (3.4), −16.67, −56.25, 29.17, −26.08, −11.12 | 28 | −7.81 (3.4), −8.33, −41.67, 27.08, −14.11, −1.51 | −0.66 | −2.26 (0.0276) | 5.12 (0.0276) |
| **HPES-Symptom Cognitive Domain (0-100 scale)** |  |  |  |  |  |  |  |
| PGIS-Overall Symptoms | 31 | −20.00 (4.8), −25.00, −80.00, 35.00, −30.05, −9.95 | 28 | −4.46 (5.1), 0.00, −55.00, 70.00, −14.61, 5.68 | −0.59 | −2.22 (0.0302) | 4.94 (0.0302) |
| PGIS-Physical Symptoms | 31 | −19.19 (4.9), −15.00, −80.00, 35.00, −28.78, −9.60 | 28 | −5.36 (5.1), 0.00, −65.00, 70.00, −16.22, 5.51 | −0.49 | −1.96 (0.0546) | 3.85 (0.0546) |
| PGIS Cognitive Symptoms | 29 | −25.52 (4.6), −25.00, −80.00, 10.00, −35.09, −15.95 | 30 | −0.17 (4.5), 0.00, −50.00, 70.00, −9.29, 8.96 | −1.04 | −3.93 (0.0002) | 15.41 (0.0002) |

ANOVA = analysis of variance; CI = confidence interval; ESE = effect size estimate; F = F-statistic; HPES = Hypoparathyroidism Patient Experience Scale; LS = least square; PGIS = patient global impression of severity; SD = standard deviation; SE = standard error.

**45B4**

### **Table S2:** Phase 2 Study HPES-Symptom: Ability to Detect Change by HP-Interference Items

| Score/Group | Improved | | No Change/Worsening | |  | | |
| --- | --- | --- | --- | --- | --- | --- | --- |
|  | n | LS Mean (SE), Median, Min, Max, Lower 95% CI, Upper 95% CI | n | LS Mean (SE), Median, Min, Max, Lower 95% CI, Upper 95% CI | Cohen’s *d* | t-Statistic (*P* Value) | ANOVA F-Statistic (*P* Value) |
| HPES-Symptom Total (0-100 scale) |  |  |  |  |  |  |  |
| HP Interference-Quality of life | 27 | −25.29 (3.1), −24.17, −68.13, 5.00, −32.18, −18.41 | 29 | −0.93 (3.0), −2.50, −26.25, 39.17, −6.69, 4.84 | −1.61 | −5.60 (< 0.0001) | 31.32 (< 0.0001) |
| HP Interference-Physical functioning | 28 | −22.88 (3.3), −20.52, −68.13, 8.13, −29.79, −15.97 | 28 | −2.47 (3.3), −2.29, −36.88, 39.17, −9.23, 4.29 | −1.17 | −4.33 (< 0.0001) | 18.76 (< 0.0001) |
| HP Interference-Daily functioning | 30 | −23.42 (3.1), −21.56, −68.13, 8.13, −30.06, −16.77 | 26 | −0.28 (3.3), −2.81, −25.63, 39.17, −6.52, 5.96 | −1.50 | −5.15 (< 0.0001) | 26.56 (< 0.0001) |
| HP Interference-Social functioning | 25 | −24.28 (3.5), −23.33, −68.13, 19.58, −32.31, −16.26 | 31 | −3.31 (3.1), −3.33, −35.21, 39.17, −9.10, 2.47 | −1.33 | −4.46 (< 0.0001) | 19.88 (< 0.0001) |
| HP Interference-Emotional well-being | 25 | −25.95 (3.3), −24.17, −68.13, 19.58, −33.58, −18.32 | 31 | −1.97 (3.0), −3.13, −26.25, 39.17, −7.35, 3.41 | −1.63 | −5.42 (< 0.0001) | 29.33 (< 0.0001) |
| HPES-Symptom Physical Domain (0-100 scale) |  |  |  |  |  |  |  |
| HP Interference-Quality of life | 27 | −22.07 (3.2), −20.83, −56.25, 25.00, −28.60, −15.54 | 29 | −4.96 (3.1), −6.25, −41.67, 29.17, −11.23, 1.32 | −1.04 | −3.88 (0.0003) | 15.03 (0.0003) |
| HP Interference-Physical functioning | 28 | −20.76 (3.2), −18.75, −56.25, 25.00, −27.66, −13.86 | 28 | −5.65 (3.2), −5.21, −35.42, 29.17, −11.93, 0.62 | −0.93 | −3.32 (0.0016) | 11.05 (0.0016) |
| HP Interference-Daily functioning | 30 | −20.83 (3.0), −19.79, −56.25, 25.00, −27.42, −14.25 | 26 | −4.41 (3.3), −7.29, −31.25, 29.17, −10.67, 1.86 | −1.06 | −3.67 (0.0006) | 13.49 (0.0006) |
| HP Interference-Social functioning | 25 | −21.17 (3.4), −20.83, −56.25, 29.17, −28.89, −13.44 | 31 | −6.79 (3.1), −8.33, −35.42, 27.08, −12.60, −0.97 | −0.91 | −3.11 (0.0030) | 9.69 (0.0030) |
| HP Interference-Emotional well-being | 25 | −20.50 (3.5), −18.75, −56.25, 29.17, −28.05, −12.95 | 31 | −7.33 (3.1), −6.25, −41.67, 27.08, −13.45, −1.20 | −0.79 | −2.81 (0.0068) | 7.91 (0.0068) |
| HPES-Symptom Cognitive Domain (0-100 scale) |  |  |  |  |  |  |  |
| HP Interference-Quality of life | 29 | −28.28 (4.3), −25.00, −80.00, 10.00, −37.76, −18.79 | 30 | 2.50 (4.2), 0.00, −30.00, 70.00, −5.45, 10.45 | −1.45 | −5.10 (< 0.0001) | 26.06 (< 0.0001) |
| HP Interference-Physical functioning | 31 | −24.68 (4.5), −25.00, −80.00, 10.00, −33.74, −15.62 | 28 | 0.71 (4.7), 0.00, −55.00, 70.00, −8.94, 10.37 | −1.02 | −3.93 (0.0002) | 15.43 (0.0002) |
| HP Interference-Daily functioning | 33 | −25.61 (4.1), −25.00, −80.00, 10.00, −34.36, −16.86 | 26 | 3.85 (4.6), 0.00, −30.00, 70.00, −5.15, 12.85 | −1.32 | −4.75 (< 0.0001) | 22.54 (< 0.0001) |
| HP Interference-Social functioning | 27 | −27.22 (4.7), −25.00, −80.00, 10.00, −37.76, −16.68 | 32 | −0.31 (4.3), 0.00, −35.00, 70.00, −8.36, 7.74 | −1.21 | −4.22 (< 0.0001) | 17.82 (< 0.0001) |
| HP Interference-Emotional well-being | 28 | −30.36 (4.2), −30.00, −80.00, 10.00, −39.80, −20.92 | 31 | 3.39 (4.0), 0.00, −25.00, 70.00, −3.88, 10.65 | −1.70 | −5.86 (< 0.0001) | 34.39 (< 0.0001) |

ANOVA = analysis of variance; CI = Confidence interval; ESE = effect size estimate; F = F-statistic; HP = hypoparathyroidism; HPES = Hypoparathyroidism Patient Experience Scale; LS = least square; SD = standard deviation; SE = standard error.

HPES Symptom - Threshold for Meaningful Within-Patient Change (Responder Definition)

### **Table S3:** Phase 2 Study Interpretation of Change–HPES-Symptom Total

| Score/Methods | Change in HPES-Symptom From Screening to Visit 3 | | | | |
| --- | --- | --- | --- | --- | --- |
| HPES-Symptom Total (0‑100 scale) |  |  |  |  |  |
| Anchor-Based Methods | n | Mean (SD) | Q1, Median, Q3 | 10th, 90th Percentile | Min, Max |
| Total Sample | 56 | −12.7 (20.28) | −24.9, −11.7, −1.7 | −38.3, 11.3 | −68.1, 39.2 |
| PRIMARY: By PGIS-Overall Symptoms |  |  |  |  |  |
| 5-point improvement | 0 | - | - | - | - |
| 4-point improvement | 1 | −15.4 (-) | −15.4, −15.4, −15.4 | −15.4, −15.4 | −15.4, −15.4 |
| 3-point improvement | 3 | −41.2 (25.39) | −68.1, −37.7, −17.7 | −68.1, −17.7 | −68.1, −17.7 |
| 2-point improvement | 8 | −23.4 (16.06) | −32.7, −25.4, −9.5 | −49.6, −2.1 | −49.6, −2.1 |
| 1-point improvement | 17 | −17.3 (16.21) | −23.3, −14.6, −6.7 | −47.1, −3.3 | −49.6, 11.3 |
| No change | 17 | −10.1 (14.78) | −21.9, −3.1, 0.0 | −36.9, 5.0 | −38.3, 8.1 |
| 1-point worsening | 8 | 6.5 (22.10) | −7.3, 5.6, 22.8 | −29.2, 39.2 | −29.2, 39.2 |
| 2-point worsening | 1 | 7.3 (-) | 7.3, 7.3, 7.3 | 7.3, 7.3 | 7.3, 7.3 |
| 3-point worsening | 1 | 22.1 (-) | 22.1, 22.1, 22.1 | 22.1, 22.1 | 22.1, 22.1 |
| 4-point worsening | 0 | - | - | - | - |
| 5-point worsening | 0 | - | - | - | - |
| By CGIS Cognitive Symptoms |  |  |  |  |  |
| 5-point improvement | 0 | - | - | - | - |
| 4-point improvement | 1 | −36.9 (-) | −36.9, −36.9, −36.9 | −36.9, −36.9 | −36.9, −36.9 |
| 3-point improvement | 1 | −37.7 (-) | −37.7, −37.7, −37.7 | −37.7, −37.7 | −37.7, −37.7 |
| 2-point improvement | 4 | −26.2 (18.19) | −39.9, −24.0, −12.5 | −49.6, −7.3 | −49.6, −7.3 |
| 1-point improvement | 16 | −19.3 (20.07) | −25.9, −17.7, −3.8 | −49.6, 0.0 | −68.1, 13.3 |
| No change | 16 | −5.8 (19.29) | −16.1, −4.9, 4.6 | −29.2, 22.1 | −47.1, 26.0 |
| 1-point worsening | 9 | 2.4 (16.30) | −6.7, −2.1, 7.3 | −15.6, 39.2 | −15.6, 39.2 |
| 2-point worsening | 1 | 0.0 (-) | 0.0, 0.0, 0.0 | 0.0, 0.0 | 0.0, 0.0 |
| 3-point worsening | 0 | - | - | - | - |
| 4-point worsening | 0 | - | - | - | - |
| 5-point worsening | 0 | - | - | - | - |
| By HP Interference-Quality of Life |  |  |  |  |  |
| 4-point improvement | 1 | −68.1 (-) | −68.1, −68.1, −68.1 | −68.1, −68.1 | −68.1, −68.1 |
| 3-point improvement | 3 | −42.9 (5.91) | −49.6, −40.8, −38.3 | −49.6, −38.3 | −49.6, −38.3 |
| 2-point improvement | 7 | −30.5 (16.08) | −47.1, −36.9, −14.6 | −49.6, −12.1 | −49.6, −12.1 |
| 1-point improvement | 16 | −17.0 (12.13) | −26.6, −19.8, −4.2 | −30.2, −2.1 | −35.2, 5.0 |
| No change | 19 | −7.7 (10.41) | −15.6, −5.6, 0.0 | −23.3, 8.1 | −26.3, 11.3 |
| 1-point worsening | 7 | 8.8 (11.67) | −2.1, 7.3, 19.6 | −6.7, 26.0 | −6.7, 26.0 |
| 2-point worsening | 2 | 30.6 (12.08) | 22.1, 30.6, 39.2 | 22.1, 39.2 | 22.1, 39.2 |
| 3-point worsening | 1 | −3.8 (-) | −3.8, −3.8, −3.8 | −3.8, −3.8 | −3.8, −3.8 |
| 4-point worsening | 0 | - | - | - | - |
| By Standard of Care |  |  |  |  |  |
| On SOC | 10 | 3.7 (12.75) | −3.3, −0.6, 11.3 | −10.7, 22.8 | −17.7, 26.0 |
| Off SOC | 46 | −16.2 (19.94) | −27.5, −15.5, −3.3 | −40.8, 5.0 | −68.1, 39.2 |
| Distribution-Based Methods |  | Estimate |  |  |  |
| Half-SD |  | 11.41 |  |  |  |
| SEM |  | 10.32 |  |  |  |

HPES = Hypoparathyroidism Patient Experience Scale; CGIS = clinical global impression of severity; HP = hypoparathyroidism; PGIS = patient global impression of severity; SEM = standard error of measurement; SOC = standard of care.

Note: Gold highlights the primary anchor level and blue highlights supportive anchor levels.

### **Table S4:** Phase 2 Study Interpretation of Change–HPES-Symptom Physical

| Score/Methods | Change in HPES-Symptom From Screening to Visit 3 | | | | |
| --- | --- | --- | --- | --- | --- |
| HPES-Symptom Physical Domain (0-100 scale) |  |  |  |  |  |
| Anchor-Based Methods | n | Mean (SD) | Q1, Median, Q3 | 10th, 90th Percentile | Min, Max |
| Total Sample | 56 | −13.2 (18.49) | −27.1, −12.5, −3.1 | −35.4, 8.3 | −56.3, 29.2 |
| PRIMARY By PGIS-Physical Symptoms |  |  |  |  |  |
| 5-point improvement | 0 | - | - | - | - |
| 4-point improvement | 1 | −56.3 (-) | −56.3, −56.3, −56.3 | −56.3, −56.3 | −56.3, −56.3 |
| 3-point improvement | 1 | −20.8 (-) | −20.8, −20.8, −20.8 | −20.8, −20.8 | −20.8, −20.8 |
| 2-point improvement | 11 | −26.1 (15.85) | −35.4, −25.0, −10.4 | −41.7, −6.3 | −54.2, −4.2 |
| 1-point improvement | 15 | −16.7 (10.56) | −29.2, −16.7, −8.3 | −29.2, −6.3 | −31.3, 0.0 |
| No change | 21 | −8.0 (17.58) | −16.7, −8.3, 0.0 | −31.3, 8.3 | −41.7, 29.2 |
| 1-point worsening | 5 | 5.0 (18.20) | −12.5, 8.3, 16.7 | −14.6, 27.1 | −14.6, 27.1 |
| 2-point worsening | 1 | 14.6 (-) | 14.6, 14.6, 14.6 | 14.6, 14.6 | 14.6, 14.6 |
| 3-point worsening | 0 | - | - | - | - |
| 4-point worsening | 0 | - | - | - | - |
| 5-point worsening | 1 | 4.2 (-) | 4.2, 4.2, 4.2 | 4.2, 4.2 | 4.2, 4.2 |
| By PGIS-Overall Symptoms |  |  |  |  |  |
| 5-point improvement | 0 | - | - | - | - |
| 4-point improvement | 1 | −20.8 (-) | −20.8, −20.8, −20.8 | −20.8, −20.8 | −20.8, −20.8 |
| 3-point improvement | 3 | −34.0 (22.95) | −56.3, −35.4, −10.4 | −56.3, −10.4 | −56.3, −10.4 |
| 2-point improvement | 8 | −27.3 (17.55) | −38.5, −30.2, −11.5 | −54.2, −4.2 | −54.2, −4.2 |
| 1-point improvement | 17 | −19.4 (9.57) | −29.2, −16.7, −12.5 | −31.3, −8.3 | −37.5, −6.3 |
| No change | 17 | −8.9 (15.41) | −18.8, −8.3, 0.0 | −31.3, 6.3 | −41.7, 25.0 |
| 1-point worsening | 8 | 8.1 (15.69) | −6.3, 8.3, 21.9 | −12.5, 29.2 | −12.5, 29.2 |
| 2-point worsening | 1 | 14.6 (-) | 14.6, 14.6, 14.6 | 14.6, 14.6 | 14.6, 14.6 |
| 3-point worsening | 1 | 4.2 (-) | 4.2, 4.2, 4.2 | 4.2, 4.2 | 4.2, 4.2 |
| 4-point worsening | 0 | - | - | - | - |
| 5-point worsening | 0 | - | - | - | - |
| By CGIS Overall Symptoms |  |  |  |  |  |
| 5-point improvement | 0 | - | - | - | - |
| 4-point improvement | 0 | - | - | - | - |
| 3-point improvement | 0 | - | - | - | - |
| 2-point improvement | 8 | −26.3 (18.19) | −38.5, −17.7, −15.6 | −56.3, −10.4 | −56.3, −10.4 |
| 1-point improvement | 22 | −14.4 (17.72) | −29.2, −14.6, −6.3 | −35.4, 8.3 | −37.5, 29.2 |
| No change | 14 | −5.1 (16.96) | −8.3, −2.1, 4.2 | −31.3, 8.3 | −41.7, 27.1 |
| 1-point worsening | 4 | −1.6 (10.81) | −7.3, −6.3, 4.2 | −8.3, 14.6 | −8.3, 14.6 |
| 2-point worsening | 0 | - | - | - | - |
| 3-point worsening | 0 | - | - | - | - |
| 4-point worsening | 0 | - | - | - | - |
| 5-point worsening | 0 | - | - | - | - |
| By CGIS Physical Symptoms |  |  |  |  |  |
| 5-point improvement | 0 | - | - | - | - |
| 4-point improvement | 0 | - | - | - | - |
| 3-point improvement | 0 | - | - | - | - |
| 2-point improvement | 6 | −28.1 (21.11) | −54.2, −16.7, −14.6 | −56.3, −10.4 | −56.3, −10.4 |
| 1-point improvement | 21 | −16.3 (15.57) | −29.2, −14.6, −6.3 | −35.4, 2.1 | −37.5, 16.7 |
| No change | 16 | −6.5 (18.30) | −18.8, −7.3, 0.0 | −31.3, 27.1 | −41.7, 29.2 |
| 1-point worsening | 5 | 2.1 (9.43) | −6.3, 4.2, 6.3 | −8.3, 14.6 | −8.3, 14.6 |
| 2-point worsening | 0 | - | - | - | - |
| 3-point worsening | 0 | - | - | - | - |
| 4-point worsening | 0 | - | - | - | - |
| 5-point worsening | 0 | - | - | - | - |
| By HP Interference-Quality of Life |  |  |  |  |  |
| 4-point improvement | 1 | −56.3 (-) | −56.3, −56.3, −56.3 | −56.3, −56.3 | −56.3, −56.3 |
| 3-point improvement | 3 | −29.2 (12.50) | −41.7, −29.2, −16.7 | −41.7, −16.7 | −41.7, −16.7 |
| 2-point improvement | 7 | −31.0 (11.69) | −35.4, −29.2, −20.8 | −54.2, −18.8 | −54.2, −18.8 |
| 1-point improvement | 16 | −14.7 (14.64) | −24.0, −13.5, −8.3 | −35.4, −4.2 | −35.4, 25.0 |
| No change | 19 | −11.4 (13.86) | −16.7, −12.5, 0.0 | −37.5, 6.3 | −41.7, 8.3 |
| 1-point worsening | 7 | 10.4 (15.31) | −4.2, 14.6, 27.1 | −8.3, 29.2 | −8.3, 29.2 |
| 2-point worsening | 2 | 6.2 (2.95) | 4.2, 6.3, 8.3 | 4.2, 8.3 | 4.2, 8.3 |
| 3-point worsening | 1 | −12.5 (-) | −12.5, −12.5, −12.5 | −12.5, −12.5 | −12.5, −12.5 |
| 4-point worsening | 0 | - | - | - | - |
| By Standard of Care |  |  |  |  |  |
| On SOC | 10 | −0.6 (16.64) | −12.5, −7.3, 6.3 | −14.6, 28.1 | −16.7, 29.2 |
| Off SOC | 46 | −15.9 (17.88) | −29.2, −15.6, −6.3 | −37.5, 8.3 | −56.3, 25.0 |
| Distribution-Based Methods |  | Estimate |  |  |  |
| Half-SD |  | 11.25 |  |  |  |
| SEM |  | 9.82 |  |  |  |

HPES = Hypoparathyroidism Patient Experience Scale; CGIS = clinical global impression of severity; HP = hypoparathyroidism; PGIS = patient global impression of severity; SEM = standard error of measurement; SOC = standard of care.

Note: Gold highlights the primary anchor level and blue highlights supportive anchor levels.

### **Table S5.** Phase 2 Study Interpretation of Change – HPES-Symptom Cognitive

| Score/Methods | Change in HPES-Symptom From Screening to Visit 3 | | | | |
| --- | --- | --- | --- | --- | --- |
| HPES-Symptom Cognitive Domain (0-100 scale) |  |  |  |  |  |
| Anchor-Based Methods | n | Mean (SD) | Q1, Median, Q3 | 10th, 90th Percentile | Min, Max |
| Total Sample | 59 | −12.6 (27.70) | −30.0, −5.0, 0.0 | −50.0, 10.0 | −80.0, 70.0 |
| PRIMARY By PGIS Cognitive Symptoms |  |  |  |  |  |
| 5-point improvement | 0 | - | - | - | - |
| 4-point improvement | 1 | −80.0 (-) | −80.0, −80.0, −80.0 | −80.0, −80.0 | −80.0, −80.0 |
| 3-point improvement | 4 | −47.5 (19.36) | −62.5, −47.5, −32.5 | −70.0, −25.0 | −70.0, −25.0 |
| 2-point improvement | 9 | −30.0 (20.77) | −45.0, −25.0, −25.0 | −65.0, 0.0 | −65.0, 0.0 |
| 1-point improvement | 15 | −13.3 (20.41) | −25.0, −10.0, 0.0 | −35.0, 10.0 | −65.0, 10.0 |
| No change | 15 | −11.7 (18.87) | −25.0, 0.0, 0.0 | −40.0, 10.0 | −50.0, 10.0 |
| 1-point worsening | 10 | −1.0 (14.30) | 0.0, 0.0, 5.0 | −25.0, 15.0 | −30.0, 20.0 |
| 2-point worsening | 4 | 35.0 (25.50) | 17.5, 30.0, 52.5 | 10.0, 70.0 | 10.0, 70.0 |
| 3-point worsening | 0 | - | - | - | - |
| 4-point worsening | 1 | 40.0 (-) | 40.0, 40.0, 40.0 | 40.0, 40.0 | 40.0, 40.0 |
| 5-point worsening | 0 | - | - | - | - |
| By CGIS Cognitive Symptoms |  |  |  |  |  |
| 5-point improvement | 0 | - | - | - | - |
| 4-point improvement | 1 | −55.0 (-) | −55.0, −55.0, −55.0 | −55.0, −55.0 | −55.0, −55.0 |
| 3-point improvement | 1 | −40.0 (-) | −40.0, −40.0, −40.0 | −40.0, −40.0 | −40.0, −40.0 |
| 2-point improvement | 5 | −19.0 (19.17) | −25.0, −25.0, 0.0 | −45.0, 0.0 | −45.0, 0.0 |
| 1-point improvement | 16 | −21.3 (25.33) | −27.5, −22.5, 0.0 | −70.0, 10.0 | −80.0, 10.0 |
| No change | 17 | −7.9 (28.78) | −25.0, 0.0, 10.0 | −50.0, 25.0 | −65.0, 40.0 |
| 1-point worsening | 9 | 11.1 (27.13) | 0.0, 5.0, 10.0 | −25.0, 70.0 | −25.0, 70.0 |
| 2-point worsening | 2 | −7.5 (10.61) | −15.0, −7.5, 0.0 | −15.0, 0.0 | −15.0, 0.0 |
| 3-point worsening | 0 | - | - | - | - |
| 4-point worsening | 0 | - | - | - | - |
| 5-point worsening | 0 | - | - | - | - |
| By HP Interference-Quality of Life |  |  |  |  |  |
| 4-point improvement | 1 | −80.0 (-) | −80.0, −80.0, −80.0 | −80.0, −80.0 | −80.0, −80.0 |
| 3-point improvement | 3 | −56.7 (18.93) | −70.0, −65.0, −35.0 | −70.0, −35.0 | −70.0, −35.0 |
| 2-point improvement | 8 | −26.3 (28.00) | −50.0, −25.0, 0.0 | −65.0, 5.0 | −65.0, 5.0 |
| 1-point improvement | 17 | −21.2 (18.07) | −30.0, −25.0, 0.0 | −50.0, 0.0 | −50.0, 10.0 |
| No change | 19 | −3.9 (17.21) | −25.0, 0.0, 10.0 | −25.0, 20.0 | −30.0, 35.0 |
| 1-point worsening | 8 | 4.4 (12.08) | −2.5, 5.0, 10.0 | −15.0, 25.0 | −15.0, 25.0 |
| 2-point worsening | 2 | 55.0 (21.21) | 40.0, 55.0, 70.0 | 40.0, 70.0 | 40.0, 70.0 |
| 3-point worsening | 1 | 5.0 (-) | 5.0, 5.0, 5.0 | 5.0, 5.0 | 5.0, 5.0 |
| 4-point worsening | 0 | - | - | - | - |
| By Standard of Care |  |  |  |  |  |
| On SOC | 10 | 8.0 (15.85) | 0.0, 10.0, 10.0 | −12.5, 30.0 | −25.0, 35.0 |
| Off SOC | 49 | −16.8 (27.81) | −30.0, −15.0, 0.0 | −55.0, 10.0 | −80.0, 70.0 |
| Distribution-Based Methods |  | Estimate |  |  |  |
| Half-SD |  | 15.09 |  |  |  |
| SEM |  | 15.99 |  |  |  |

HPES = Hypoparathyroidism Patient Experience Scale; CGIS = clinical global impression of severity; HP = hypoparathyroidism; PGIS = patient global impression of severity; SEM = standard error of measurement; SOC = standard of care.

Note: Gold highlights the primary anchor level and blue highlights supportive anchor levels.

### **Fig. S1:** Cumulative Distribution Function of Change in HPES Symptom Total Score from Screening to Visit 3, by change in PGIS-Overall Symptoms

HPES = Hypoparathyroid Patient Experience Scale; PGIS = patient global impression of severity.

Note: PGIS items used a 6-point response scale: 0 = No noticeable, 1 = Very mild, 2 = Mild, 3 = Moderate, 4 = Severe, 5 = Very severe.

### **Fig. S2:** Probability Density Function of Change in HPES Symptom Total Score from Screening to Visit 3, by Change in PGIS-Overall Symptoms


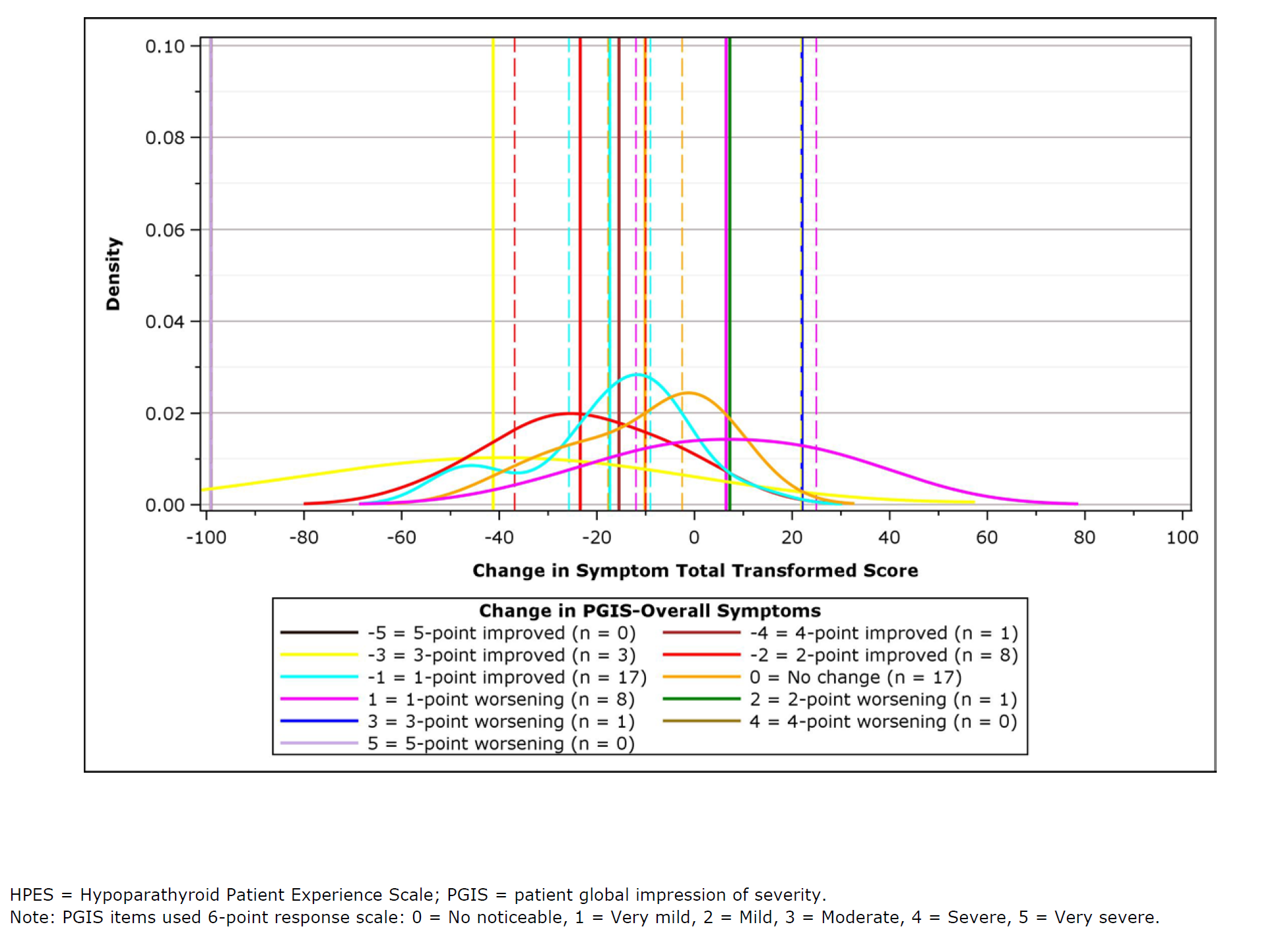


### **Fig. S3**. Cumulative Distribution Function of Change in HPES Symptom Physical Score from Screening to Visit 3, by Change in PGIS-Physical Symptom

HPES = Hypoparathyroid Patient Experience Scale; PGIS = patient global impression of severity.

Note: PGIS items used a 6-point response scale: 0 = No noticeable, 1 = Very mild, 2 = Mild, 3 = Moderate, 4 = Severe, 5 = Very severe.

### **Fig. S4.** Probability Density Function of Change in HPES Symptom Physical Score from Screening to Visit 3, by Change in PGIS-Physical Symptoms


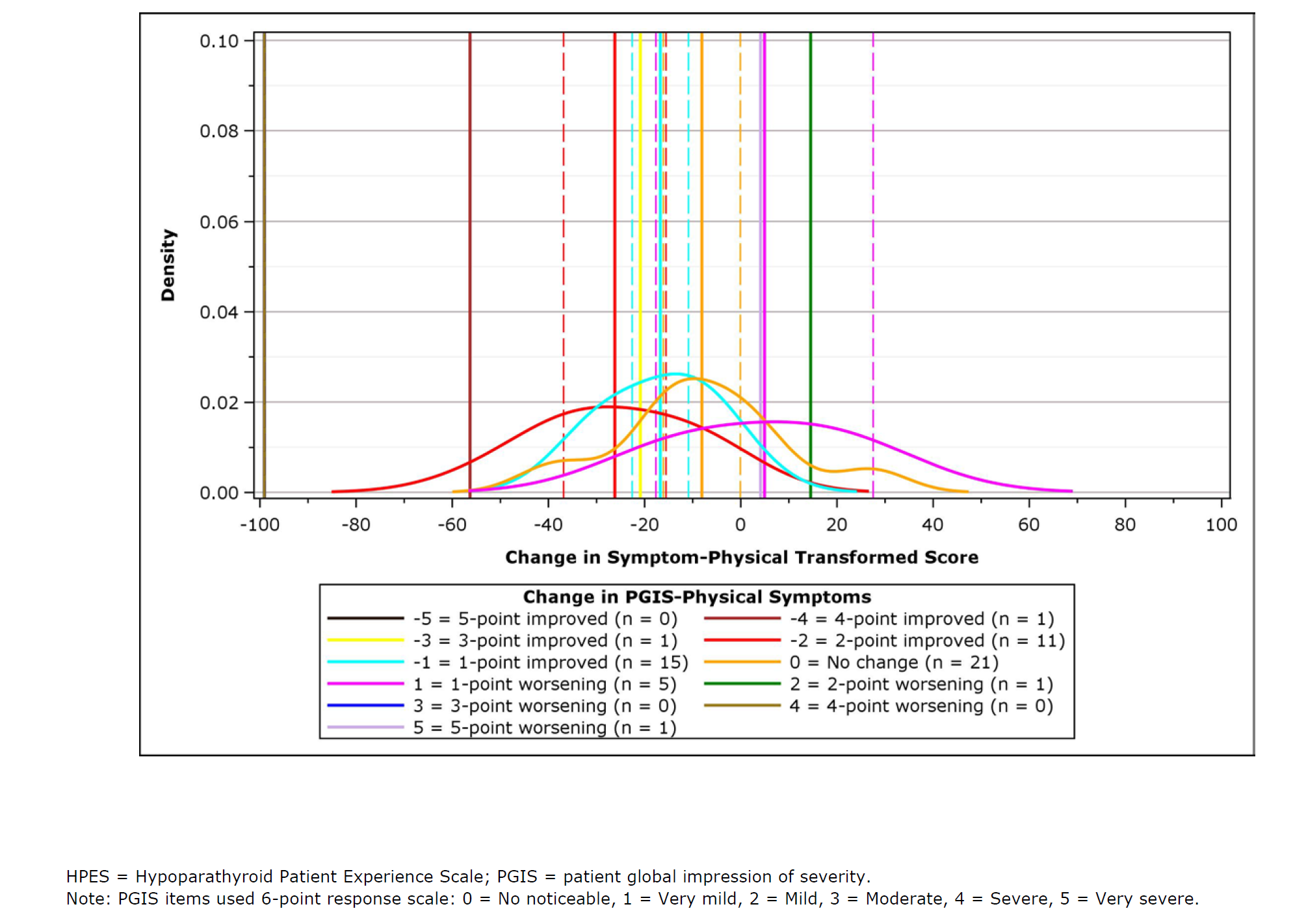


### **Fig. S5.** Cumulative Distribution Function of Change in HPES Symptom Cognitive Score from Screening to Visit 3, by Change in PGIS-Cognitive Symptoms

HPES = Hypoparathyroid Patient Experience Scale; PGIS = patient global impression of severity.

Note: PGIS items used a 6-point response scale: 0 = No noticeable, 1 = Very mild, 2 = Mild, 3 = Moderate, 4 = Severe, 5 = Very severe.

### **Fig. S6.** Probability Density Function of Change in HPES Symptom Cognitive Score from Screening to Visit 3, by Change in PGIS-Cognitive Symptoms


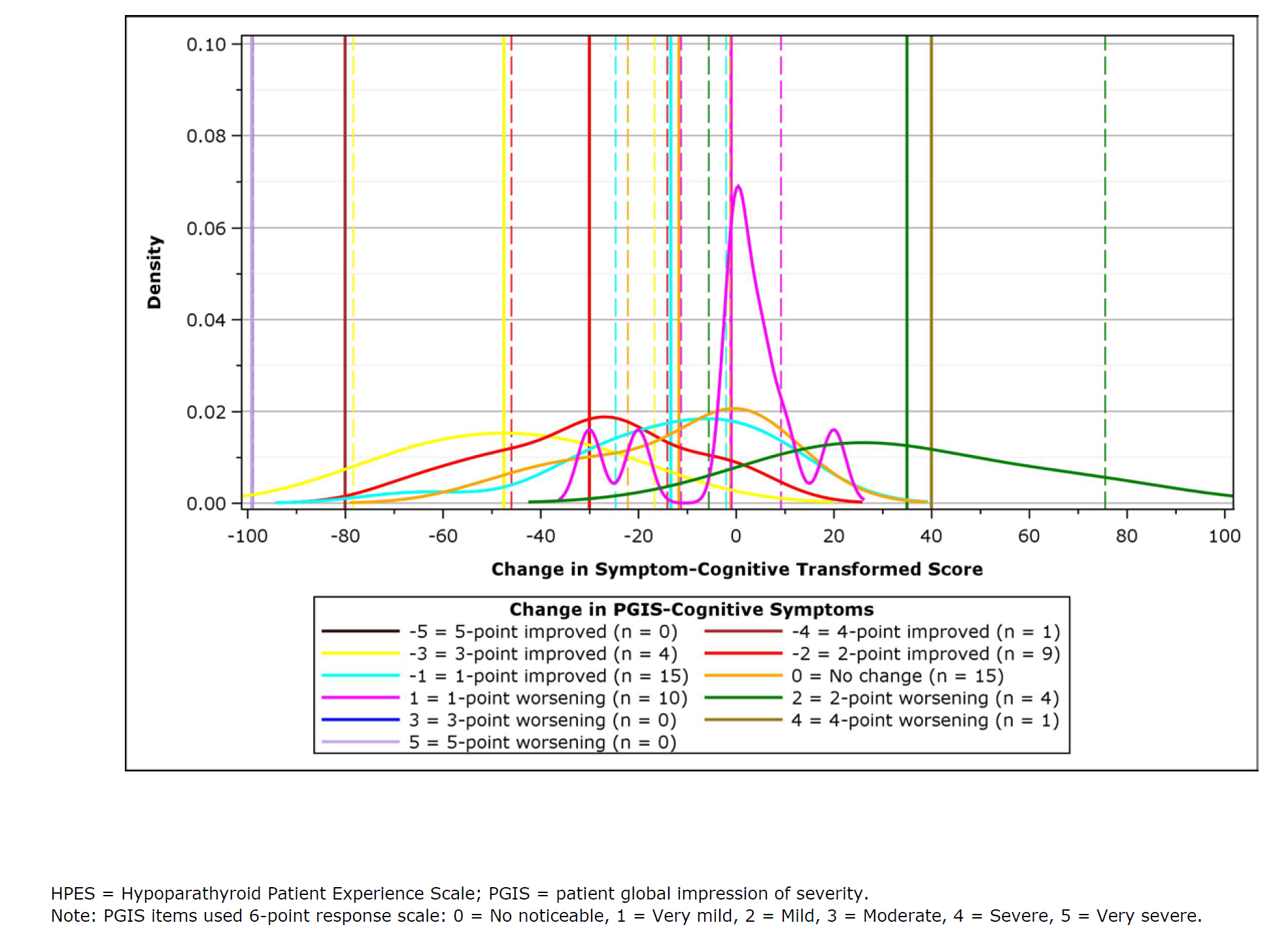


HPES Impact - Ability to Detect Change

### **Table S6.** Phase 2 Study HPES-Impact: Ability to Detect Change by PGIS

| **Score/Group** | **Improved** | | **No Change/Worsening** | |  | | |
| --- | --- | --- | --- | --- | --- | --- | --- |
|  | **n** | **LS Mean (SE), Median, Min, Max, Lower 95% CI, Upper 95% CI** | **n** | **LS Mean (SE), Median, Min, Max, Lower 95% CI, Upper 95% CI** | **Cohen’s *d*** | **t-statistic (*P* Value)** | **ANOVA F-Statistic (*P* Value)** |
| **HPES-Impact Total (0-100 scale)** |  |  |  |  |  |  |  |
| PGIS-Overall Symptoms | 30 | −19.99 (3.1), −15.38, −57.63, 1.72, −26.54, −13.45 | 27 | −0.68 (3.2), 0.00, −41.72, 32.41, −6.96, 5.59 | −1.22 | −4.34 (< 0.0001) | 18.85 (< 0.0001) |
| PGIS-Physical Symptoms | 30 | −17.26 (3.3), −12.62, −57.63, 6.96, −24.10, −10.42 | 27 | −3.72 (3.5), 0.00, −41.94, 32.41, −10.84, 3.40 | −0.75 | −2.81 (0.0069) | 7.89 (0.0069) |
| PGIS Cognitive Symptoms | 29 | −17.80 (3.3), −15.29, −57.63, 15.83, −25.32, −10.28 | 28 | −3.64 (3.4), −1.64, −41.72, 32.41, −9.87, 2.58 | −0.88 | −2.96 (0.0045) | 8.78 (0.0045) |
| **HPES-Impact Physical Functioning Domain (0-100 scale)** |  |  |  |  |  |  |  |
| PGIS-Overall Symptoms | 31 | −23.71 (3.4), −20.00, −75.00, 10.00, −31.18, −16.24 | 27 | 1.67 (3.6), 0.00, −35.00, 40.00, −5.12, 8.45 | −1.48 | −5.09 (< 0.0001) | 25.89 (< 0.0001) |
| PGIS-Physical Symptoms | 31 | −19.84 (3.8), −15.00, −75.00, 10.00, −27.96, −11.71 | 27 | −2.78 (4.1), 0.00, −45.00, 40.00, −10.71, 5.16 | −0.85 | −3.06 (0.0034) | 9.34 (0.0034) |
| PGIS Cognitive Symptoms | 29 | −18.10 (4.1), −15.00, −75.00, 35.00, −27.43, −8.78 | 29 | −5.69 (4.1), 0.00, −50.00, 40.00, −13.00, 1.62 | −0.65 | −2.15 (0.0362) | 4.61 (0.0362) |
| **HPES-Impact Daily Life Domain (0-100 scale)** |  |  |  |  |  |  |  |
| PGIS-Overall Symptoms | 30 | −22.66 (3.3), −23.44, −64.29, 6.25, −29.80, −15.53 | 28 | −2.33 (3.5), 0.00, −50.00, 32.14, −9.07, 4.41 | −1.17 | −4.23 (< 0.0001) | 17.89 (< 0.0001) |
| PGIS-Physical Symptoms | 30 | −18.38 (3.7), −17.19, −64.29, 17.86, −25.55, −11.21 | 28 | −6.92 (3.8), 0.00, −57.14, 32.14, −15.14, 1.30 | −0.54 | −2.16 (0.0351) | 4.66 (0.0351) |
| PGIS Cognitive Symptoms | 29 | −21.11 (3.6), −21.88, −64.29, 21.43, −29.69, −12.52 | 29 | −4.59 (3.6), −3.13, −34.38, 32.14, −10.39, 1.22 | −1.08 | −3.26 (0.0019) | 10.66 (0.0019) |
| **HPES-Impact Psychological Well-Being Domain (0-100 scale)** |  |  |  |  |  |  |  |
| PGIS-Overall Symptoms | 31 | −14.21 (3.9), −15.63, −62.50, 31.25, −22.23, −6.20 | 28 | −3.68 (4.1), 0.00, −62.50, 62.50, −12.10, 4.73 | −0.49 | −1.85 (0.0689) | 3.44 (0.0689) |
| PGIS-Physical Symptoms | 31 | −13.51 (3.9), −9.38, −62.50, 31.25, −21.54, −5.48 | 28 | −4.46 (4.1), 0.00, −62.50, 62.50, −13.00, 4.08 | −0.41 | −1.58 (0.1197) | 2.50 (0.1197) |
| PGIS Cognitive Symptoms | 29 | −15.19 (4.0), −9.38, −62.50, 21.88, −23.34, −7.05 | 30 | −3.44 (3.9), 0.00, −62.50, 62.50, −11.58, 4.71 | −0.54 | −2.09 (0.0413) | 4.36 (0.0413) |
| **HPES-Impact Social Life and Relationships Domain (0-100 scale)** |  |  |  |  |  |  |  |
| PGIS-Overall Symptoms | 31 | −15.73 (3.5), −10.00, −65.00, 45.00, −23.47, −7.98 | 28 | 1.38 (3.6), 0.00, −35.00, 35.00, −5.20, 7.97 | −1.01 | −3.41 (0.0012) | 11.60 (0.0012) |
| PGIS-Physical Symptoms | 31 | −13.87 (3.6), −10.00, −65.00, 45.00, −21.52, −6.22 | 28 | −0.67 (3.8), 0.00, −50.00, 35.00, −8.08, 6.74 | −0.69 | −2.53 (0.0143) | 6.38 (0.0143) |
| PGIS Cognitive Symptoms | 29 | −16.81 (3.5), −10.00, −65.00, 10.00, −24.03, −9.59 | 30 | 1.29 (3.5), 0.00, −35.00, 45.00, −5.83, 8.41 | −0.95 | −3.65 (0.0006) | 13.35 (0.0006) |

ANOVA = analysis of variance; CI = Confidence interval; ESE = effect size estimate; F = F-statistic; HPES = Hypoparathyroidism Patient Experience Scale; LS = least square; PGIS = Patient Global Impression of Severity; SD = standard deviation; SE = standard error.

### **Table S7**. Phase 2 Study HPES-Impact: Ability to Detect Change by HP-Interference Items

| **Score/Group** | **Improved** | | **No Change/Worsening** | |  | | |
| --- | --- | --- | --- | --- | --- | --- | --- |
|  | **n** | **LS Mean (SE), Median, Min, Max, Lower 95% CI, Upper 95% CI** | **n** | **LS Mean (SE), Median, Min, Max, Lower 95% CI, Upper 95% CI** | **Cohen’s *d*** | **t-statistic (*P* Value)** | **ANOVA F-Statistic (*P* Value)** |
| **HPES-Impact Total (0-100 scale)** |  |  |  |  |  |  |  |
| HP Interference-Quality of life | 29 | −23.42 (2.7), −24.00, −57.63, 7.81, −29.87, −16.98 | 28 | 2.18 (2.7), 0.00, −15.29, 32.41, −2.17, 6.52 | −2.29 | −6.70 (< 0.0001) | 44.95 (< 0.0001) |
| HP Interference-Physical functioning | 30 | −20.16 (3.0), −16.16, −57.63, 7.81, −27.04, −13.28 | 27 | −0.50 (3.2), 0.00, −28.59, 32.41, −6.21, 5.21 | −1.36 | −4.45 (< 0.0001) | 19.80 (< 0.0001) |
| HP Interference-Daily functioning | 32 | −20.22 (2.9), −16.25, −57.63, 7.81, −26.71, −13.73 | 25 | 1.15 (3.2), 0.00, −27.34, 32.41, −4.37, 6.68 | −1.60 | −4.96 (< 0.0001) | 24.58 (< 0.0001) |
| HP Interference-Social functioning | 26 | −24.14 (2.9), −24.11, −57.63, 15.83, −31.33, −16.96 | 31 | 0.30 (2.7), 0.00, −28.04, 32.41, −4.14, 4.75 | −2.02 | −6.15 (< 0.0001) | 37.77 (< 0.0001) |
| HP Interference-Emotional well-being | 28 | −22.36 (3.0), −23.09, −57.63, 15.83, −29.56, −15.16 | 29 | 0.27 (2.9), 0.00, −25.94, 32.41, −4.36, 4.90 | −1.86 | −5.46 (< 0.0001) | 29.82 (< 0.0001) |
| **HPES-Impact Physical Functioning Domain (0-100 scale)** |  |  |  |  |  |  |  |
| HP Interference-Quality of life | 29 | −24.66 (3.5), −25.00, −75.00, 25.00, −33.14, −16.17 | 29 | 0.86 (3.5), 0.00, −20.00, 40.00, −4.71, 6.43 | −1.74 | −5.15 (< 0.0001) | 26.50 (< 0.0001) |
| HP Interference-Physical functioning | 30 | −22.50 (3.7), −20.00, −75.00, 25.00, −31.06, −13.94 | 28 | −0.54 (3.8), 0.00, −35.00, 40.00, −6.85, 5.78 | −1.35 | −4.18 (0.0001) | 17.46 (0.0001) |
| HP Interference-Daily functioning | 32 | −22.19 (3.5), −17.50, −75.00, 25.00, −30.27, −14.10 | 26 | 0.77 (3.9), 0.00, −25.00, 40.00, −5.61, 7.15 | −1.45 | −4.40 (< 0.0001) | 19.39 (< 0.0001) |
| HP Interference-Social functioning | 26 | −25.77 (3.7), −27.50, −75.00, 35.00, −35.20, −16.34 | 32 | −0.63 (3.4), 0.00, −35.00, 40.00, −5.92, 4.67 | −1.71 | −5.00 (< 0.0001) | 24.99 (< 0.0001) |
| HP Interference-Emotional well-being | 28 | −22.86 (3.8), −22.50, −75.00, 35.00, −32.00, −13.71 | 30 | −1.67 (3.7), 0.00, −50.00, 40.00, −7.82, 4.48 | −1.29 | −3.99 (0.0002) | 15.91 (0.0002) |
| **HPES-Impact Daily Life Domain (0-100 scale)** |  |  |  |  |  |  |  |
| HP Interference-Quality of life | 29 | −24.72 (3.2), −25.00, −64.29, 3.13, −31.90, −17.55 | 29 | −0.97 (3.2), 0.00, −39.29, 32.14, −6.81, 4.87 | −1.55 | −5.26 (< 0.0001) | 27.67 (< 0.0001) |
| HP Interference-Physical functioning | 31 | −22.18 (3.3), −21.88, −64.29, 17.86, −29.44, −14.91 | 27 | −2.13 (3.5), 0.00, −50.00, 32.14, −8.70, 4.44 | −1.21 | −4.14 (0.0001) | 17.14 (0.0001) |
| HP Interference-Daily functioning | 33 | −23.23 (3.0), −21.88, −64.29, 17.86, −30.19, −16.26 | 25 | 0.86 (3.4), 0.00, −28.57, 32.14, −4.56, 6.28 | −1.83 | −5.29 (< 0.0001) | 28.02 (< 0.0001) |
| HP Interference-Social functioning | 27 | −26.95 (3.1), −25.00, −64.29, 21.43, −34.54, −19.36 | 31 | −0.56 (2.9), 0.00, −32.14, 32.14, −5.35, 4.23 | −2.02 | −6.19 (< 0.0001) | 38.31 (< 0.0001) |
| HP Interference-Emotional well-being | 28 | −22.64 (3.5), −23.44, −64.29, 21.43, −31.27, −14.02 | 30 | −3.71 (3.4), 0.00, −39.29, 32.14, −9.16, 1.75 | −1.30 | −3.86 (0.0003) | 14.88 (0.0003) |
| **HPES-Impact Psychological Well-Being Domain (0-100 scale)** |  |  |  |  |  |  |  |
| HP Interference-Quality of life | 29 | −22.84 (3.3), −18.75, −62.50, 3.13, −30.13, −15.56 | 30 | 3.96 (3.3), 0.00, −28.13, 62.50, −2.17, 10.09 | −1.63 | −5.78 (< 0.0001) | 33.39 (< 0.0001) |
| HP Interference-Physical functioning | 31 | −16.94 (3.7), −15.63, −62.50, 21.88, −24.89, −8.98 | 28 | −0.67 (3.9), 0.00, −34.38, 62.50, −8.38, 7.04 | −0.82 | −2.99 (0.0041) | 8.95 (0.0041) |
| HP Interference-Daily functioning | 33 | −16.86 (3.6), −12.50, −62.50, 21.88, −24.33, −9.39 | 26 | 0.48 (4.1), 0.00, −34.38, 62.50, −7.64, 8.60 | −0.86 | −3.20 (0.0022) | 10.25 (0.0022) |
| HP Interference-Social functioning | 27 | −21.30 (3.7), −18.75, −62.50, 3.13, −29.52, −13.07 | 32 | 0.98 (3.4), 0.00, −31.25, 62.50, −5.56, 7.51 | −1.23 | −4.40 (< 0.0001) | 19.34 (< 0.0001) |
| HP Interference-Emotional well-being | 28 | −23.33 (3.4), −20.31, −62.50, 0.00, −30.89, −15.76 | 31 | 3.53 (3.2), 0.00, −18.75, 62.50, −2.38, 9.44 | −1.67 | −5.78 (< 0.0001) | 33.46 (< 0.0001) |
| **HPES-Impact Social Life and Relationships Domain (0-100 scale)** |  |  |  |  |  |  |  |
| HP Interference-Quality of life | 29 | −21.47 (3.0), −20.00, −65.00, 0.00, −28.03, −14.90 | 30 | 5.79 (2.9), 0.00, −10.00, 45.00, 0.35, 11.24 | −1.87 | −6.56 (< 0.0001) | 43.04 (< 0.0001) |
| HP Interference-Physical functioning | 31 | −17.50 (3.3), −10.00, −65.00, 10.00, −24.37, −10.63 | 28 | 3.35 (3.5), 0.00, −25.00, 45.00, −3.55, 10.25 | −1.17 | −4.37 (< 0.0001) | 19.11 (< 0.0001) |
| HP Interference-Daily functioning | 33 | −17.16 (3.1), −12.50, −65.00, 10.00, −23.74, −10.58 | 26 | 4.52 (3.5), 0.00, −25.00, 45.00, −2.52, 11.56 | −1.24 | −4.57 (< 0.0001) | 20.91 (< 0.0001) |
| HP Interference-Social functioning | 27 | −20.32 (3.4), −20.00, −65.00, 10.00, −27.91, −12.74 | 32 | 3.13 (3.1), 0.00, −25.00, 45.00, −2.61, 8.86 | −1.47 | −5.13 (< 0.0001) | 26.36 (< 0.0001) |
| HP Interference-Emotional well-being | 28 | −20.63 (3.2), −20.00, −65.00, 10.00, −27.85, −13.40 | 31 | 4.15 (3.0), 0.00, −25.00, 45.00, −1.47, 9.78 | −1.62 | −5.60 (< 0.0001) | 31.33 (< 0.0001) |

ANOVA = analysis of variance; CI = Confidence interval; ESE = effect size estimate; F = F-statistic; HP = hypoparathyroidism; HPES = Hypoparathyroidism Patient Experience Scale; LS = least square; SD = standard deviation; SE = standard error.

HPES Impact - Threshold for Meaningful Within-Patient Change (Responder Definition)

### **Table S8.** Phase 2 Study Interpretation of Change – HPES-Impact Total

| **Score/Methods** | **Changes in HPES From Screening to Visit 3** | | | | |
| --- | --- | --- | --- | --- | --- |
| **HPES-Impact Total (0-100 scale)** |  |  |  |  |  |
| **Anchor-based Methods** | **n** | **Mean (SD)** | **Q1, Median, Q3** | **10th, 90th Percentile** | **Min, Max** |
| Total Sample | 57 | −10.8 (19.25) | −24.0, −7.2, 0.0 | −41.7, 7.8 | −57.6, 32.4 |
| **PRIMARY By HP Interference-Quality of life** |  |  |  |  |  |
| 4-point improvement | 1 | −52.8 (-) | −52.8, −52.8, −52.8 | −52.8, −52.8 | −52.8, −52.8 |
| 3-point improvement | 3 | −47.1 (9.12) | −57.6, −41.9, −41.7 | −57.6, −41.7 | −57.6, −41.7 |
| 2-point improvement | 8 | −32.8 (12.70) | −41.7, −29.5, −27.3 | −52.3, −13.1 | −52.3, −13.1 |
| 1-point improvement | 17 | −13.1 (10.27) | −22.2, −15.2, −6.9 | −27.3, −0.6 | −28.0, 7.8 |
| No change | 17 | −3.7 (5.88) | −7.2, −1.3, 0.0 | −13.4, 1.7 | −15.3, 1.9 |
| 1-point worsening | 8 | 8.0 (7.28) | 2.5, 5.8, 13.3 | −0.2, 20.5 | −0.2, 20.5 |
| 2-point worsening | 2 | 30.3 (2.92) | 28.3, 30.3, 32.4 | 28.3, 32.4 | 28.3, 32.4 |
| 3-point worsening | 1^a^ | 0.0 (-) | 0.0, 0.0, 0.0 | 0.0, 0.0 | 0.0, 0.0 |
| 4-point worsening | 0 | - | - | - | - |
| **By PGIS-Overall Symptoms** |  | **-** | **-** | **-** | **-** |
| 5-point improvement | 0 | - | - | - | - |
| 4-point improvement | 1 | −29.5 (-) | −29.5, −29.5, −29.5 | −29.5, −29.5 | −29.5, −29.5 |
| 3-point improvement | 4 | −37.9 (17.69) | −52.6, −40.9, −23.2 | −52.8, −17.0 | −52.8, −17.0 |
| 2-point improvement | 9 | −18.0 (15.47) | −24.2, −15.5, −8.9 | −49.2, 1.7 | −49.2, 1.7 |
| 1-point improvement | 16 | −16.0 (17.13) | −26.6, −12.7, −1.8 | −41.9, 0.0 | −57.6, 1.6 |
| No change | 17 | −6.5 (14.06) | −15.2, −1.1, 0.0 | −28.6, 7.8 | −41.7, 10.8 |
| 1-point worsening | 8 | 5.5 (13.09) | −3.4, 2.5, 10.3 | −7.2, 32.4 | −7.2, 32.4 |
| 2-point worsening | 1 | 20.5 (-) | 20.5, 20.5, 20.5 | 20.5, 20.5 | 20.5, 20.5 |
| 3-point worsening | 1 | 28.3 (-) | 28.3, 28.3, 28.3 | 28.3, 28.3 | 28.3, 28.3 |
| 4-point worsening | 0 | - | - | - | - |
| 5-point worsening | 0 | - | - | - | - |
| **By CGIS Cognitive Symptoms** |  | **-** | **-** | **-** | **-** |
| 5-point improvement | 0 | - | - | - | - |
| 4-point improvement | 1 | −28.6 (-) | −28.6, −28.6, −28.6 | −28.6, −28.6 | −28.6, −28.6 |
| 3-point improvement | 1 | −52.3 (-) | −52.3, −52.3, −52.3 | −52.3, −52.3 | −52.3, −52.3 |
| 2-point improvement | 5 | −20.9 (19.16) | −29.4, −17.0, −8.9 | −49.2, 0.0 | −49.2, 0.0 |
| 1-point improvement | 16 | −15.2 (20.03) | −25.9, −5.5, −0.9 | −52.8, 1.7 | −57.6, 4.3 |
| No change | 16 | −5.9 (14.94) | −14.3, −7.7, 0.4 | −24.0, 15.8 | −34.1, 28.3 |
| 1-point worsening | 8 | 2.8 (18.69) | −8.5, 0.7, 15.7 | −25.9, 32.4 | −25.9, 32.4 |
| 2-point worsening | 2 | 4.4 (3.60) | 1.9, 4.4, 7.0 | 1.9, 7.0 | 1.9, 7.0 |
| 3-point worsening | 0 | - | - | - | - |
| 4-point worsening | 0 | - | - | - | - |
| 5-point worsening | 0 | - | - | - | - |
| **By Standard of care** |  |  |  |  |  |
| On SOC | 8 | −0.2 (9.28) | −5.2, 0.8, 3.3 | −15.5, 15.8 | −15.5, 15.8 |
| Off SOC | 49 | −12.6 (19.95) | −25.9, −8.9, 0.0 | −41.9, 7.8 | −57.6, 32.4 |
| **Distribution-based methods** |  | **Estimate** |  |  |  |
| Half-SD |  | 11.02 |  |  |  |
| SEM |  | 11.46 |  |  |  |

HPES = Hypoparathyroidism Patient Experience Scale; CGIS = clinical global impression of severity; HP = hypoparathyroidism; PGIS = patient global impression of severity; SEM = standard error of measurement; SOC = standard of care.

^a^ One person with a change of 0 between screening and visit 3.

Note: Gold highlights the primary anchor level and blue highlights supportive anchor levels.

### **Table S9.** Phase 2 Study Interpretation of Change–HPES-Impact Physical Function

| **Score/Methods** | **Changes in HPES From Screening to Visit 3** | | | | |
| --- | --- | --- | --- | --- | --- |
| **HPES-Impact Physical Functioning Domain (0-100 scale)** |  |  |  |  |  |
| **Anchor-based Methods** | **n** | **Mean (SD)** | **Q1, Median, Q3** | **10th, 90th Percentile** | **Min, Max** |
| Total Sample | 58 | −11.9 (22.71) | −25.0, −10.0, 0.0 | −45.0, 15.0 | −75.0, 40.0 |
| **PRIMARY By HP Interference-Physical functioning** |  |  |  |  |  |
| 4-point improvement | 0 | − | − | − | − |
| 3-point improvement | 4 | −45.0 (22.73) | −60.0, −42.5, −30.0 | −75.0, −20.0 | −75.0, −20.0 |
| 2-point improvement | 9 | −30.6 (16.67) | −45.0, −35.0, −15.0 | −50.0, −10.0 | −50.0, −10.0 |
| 1-point improvement | 17 | −12.9 (21.36) | −30.0, −10.0, 0.0 | −45.0, 10.0 | −50.0, 25.0 |
| No change | 23 | −3.3 (14.59) | −15.0, 0.0, 0.0 | −20.0, 15.0 | −35.0, 35.0 |
| 1-point worsening | 4 | 8.8 (20.97) | −2.5, 0.0, 20.0 | −5.0, 40.0 | −5.0, 40.0 |
| 2-point worsening | 1 | 25.0 (-) | 25.0, 25.0, 25.0 | 25.0, 25.0 | 25.0, 25.0 |
| 3-point worsening | 0 | - | - | - | - |
| 4-point worsening | 0 | - | - | - | - |
| **By HP Interference-Quality of life** |  |  |  |  |  |
| 4-point improvement | 1 | −75.0 (-) | −75.0, −75.0, −75.0 | −75.0, −75.0 | −75.0, −75.0 |
| 3-point improvement | 3 | −41.7 (5.77) | −45.0, −45.0, −35.0 | −45.0, −35.0 | −45.0, −35.0 |
| 2-point improvement | 8 | −36.3 (16.64) | −47.5, −42.5, −25.0 | −50.0, −10.0 | −50.0, −10.0 |
| 1-point improvement | 17 | −13.2 (18.11) | −25.0, −15.0, −5.0 | −35.0, 10.0 | −45.0, 25.0 |
| No change | 18 | −5.3 (10.36) | −15.0, −2.5, 0.0 | −20.0, 10.0 | −20.0, 15.0 |
| 1-point worsening | 8 | 11.9 (16.68) | 0.0, 2.5, 25.0 | 0.0, 40.0 | 0.0, 40.0 |
| 2-point worsening | 2 | 12.5 (17.68) | 0.0, 12.5, 25.0 | 0.0, 25.0 | 0.0, 25.0 |
| 3-point worsening | 1 | 0.0 (-) | 0.0, 0.0, 0.0 | 0.0, 0.0 | 0.0, 0.0 |
| 4-point worsening | 0 | - | - | - | - |
| **By PGIS-Overall Symptoms** |  |  |  |  |  |
| 5-point improvement | 0 | - | - | - | - |
| 4-point improvement | 1 | −40.0 (-) | −40.0, −40.0, −40.0 | −40.0, −40.0 | −40.0, −40.0 |
| 3-point improvement | 4 | −46.3 (24.62) | −62.5, −47.5, −30.0 | −75.0, −15.0 | −75.0, −15.0 |
| 2-point improvement | 9 | −22.2 (19.06) | −35.0, −20.0, −10.0 | −45.0, 10.0 | −45.0, 10.0 |
| 1-point improvement | 17 | −18.2 (17.49) | −25.0, −15.0, 0.0 | −45.0, 0.0 | −50.0, 0.0 |
| No change | 17 | −2.9 (15.21) | −10.0, 0.0, 5.0 | −30.0, 15.0 | −35.0, 25.0 |
| 1-point worsening | 8 | 3.8 (14.82) | −5.0, 0.0, 7.5 | −10.0, 35.0 | −10.0, 35.0 |
| 2-point worsening | 1 | 40.0 (-) | 40.0, 40.0, 40.0 | 40.0, 40.0 | 40.0, 40.0 |
| 3-point worsening | 1 | 25.0 (-) | 25.0, 25.0, 25.0 | 25.0, 25.0 | 25.0, 25.0 |
| 4-point worsening | 0 | - | - | - | - |
| 5-point worsening | 0 | - | - | - | - |
| **By PGIS-Physical Symptoms** |  |  |  |  |  |
| 5-point improvement | 0 | - | - | - | - |
| 4-point improvement | 1 | −75.0 (-) | −75.0, −75.0, −75.0 | −75.0, −75.0 | −75.0, −75.0 |
| 3-point improvement | 1 | −40.0 (-) | −40.0, −40.0, −40.0 | −40.0, −40.0 | −40.0, −40.0 |
| 2-point improvement | 13 | −24.2 (19.35) | −45.0, −20.0, −10.0 | −45.0, −5.0 | −50.0, 10.0 |
| 1-point improvement | 16 | −11.6 (19.04) | −22.5, −2.5, 0.0 | −45.0, 10.0 | −50.0, 10.0 |
| No change | 20 | −6.3 (19.12) | −17.5, −2.5, 0.0 | −32.5, 20.0 | −45.0, 35.0 |
| 1-point worsening | 5 | −3.0 (13.04) | −10.0, 0.0, 0.0 | −20.0, 15.0 | −20.0, 15.0 |
| 2-point worsening | 1 | 40.0 (-) | 40.0, 40.0, 40.0 | 40.0, 40.0 | 40.0, 40.0 |
| 3-point worsening | 0 | - | - | - | - |
| 4-point worsening | 0 | - | - | - | - |
| 5-point worsening | 1 | 25.0 (-) | 25.0, 25.0, 25.0 | 25.0, 25.0 | 25.0, 25.0 |
| **By CGIS Physical Symptoms** |  |  |  |  |  |
| 5-point improvement | 0 | - | - | - | - |
| 4-point improvement | 0 | - | - | - | - |
| 3-point improvement | 0 | - | - | - | - |
| 2-point improvement | 7 | −30.0 (27.39) | −50.0, −20.0, −5.0 | −75.0, 0.0 | −75.0, 0.0 |
| 1-point improvement | 22 | −11.4 (20.36) | −20.0, −2.5, 0.0 | −45.0, 10.0 | −50.0, 15.0 |
| No change | 17 | −9.7 (16.34) | −15.0, −10.0, 0.0 | −35.0, 0.0 | −35.0, 35.0 |
| 1-point worsening | 4 | 16.3 (19.74) | 0.0, 12.5, 32.5 | 0.0, 40.0 | 0.0, 40.0 |
| 2-point worsening | 0 | - | - | - | - |
| 3-point worsening | 0 | - | - | - | - |
| 4-point worsening | 0 | - | - | - | - |
| 5-point worsening | 0 | - | - | - | - |
| **By Standard of care** |  |  |  |  |  |
| On SOC | 9 | −0.6 (17.04) | −15.0, 0.0, 0.0 | −20.0, 35.0 | −20.0, 35.0 |
| Off SOC | 49 | −14.0 (23.14) | −35.0, −10.0, 0.0 | −45.0, 10.0 | −75.0, 40.0 |
| **Distribution-based methods** |  | **Estimate** |  |  |  |
| Half-SD |  | 13.37 |  |  |  |
| SEM |  | 13.82 |  |  |  |

HPES = Hypoparathyroidism Patient Experience Scale; CGIS = clinical global impression of severity; HP = hypoparathyroidism; PGIS = patient global impression of severity; SEM = standard error of measurement; SOC = standard of care.

Note: Gold highlights the primary anchor level and blue highlights supportive anchor levels.

### **Table S10.** Phase 2 Study Interpretation of Change – HPES-Impact Daily Life

| **Score/Methods** | **Changes in HPES From Screening to Visit 3** | | | | |
| --- | --- | --- | --- | --- | --- |
| **HPES-Impact Daily Life Domain (0-100 scale)** |  |  |  |  |  |
| **Anchor-based Methods** | **n** | **Mean (SD)** | **Q1, Median, Q3** | **10th, 90th Percentile** | **Min, Max** |
| Total Sample | 58 | −12.8 (20.83) | −25.0, −6.7, 0.0 | −47.8, 9.4 | −64.3, 32.1 |
| **PRIMARY By HP Interference-Daily functioning** |  |  |  |  |  |
| 4-point improvement | 0 | - | - | - | - |
| 3-point improvement | 4 | −50.9 (10.26) | −57.1, −50.0, −44.6 | −64.3, −39.3 | −64.3, −39.3 |
| 2-point improvement | 9 | −31.3 (14.77) | −34.4, −28.1, −21.9 | −57.1, −15.6 | −57.1, −15.6 |
| 1-point improvement | 20 | −14.0 (16.13) | −25.0, −10.9, −3.1 | −35.7, 3.1 | −47.8, 17.9 |
| No change | 19 | −2.2 (11.27) | −3.1, 0.0, 0.0 | −25.0, 9.4 | −28.6, 21.4 |
| 1-point worsening | 5 | 8.3 (15.46) | −3.1, 7.1, 12.5 | −7.1, 32.1 | −7.1, 32.1 |
| 2-point worsening | 1 | 21.9 (-) | 21.9, 21.9, 21.9 | 21.9, 21.9 | 21.9, 21.9 |
| 3-point worsening | 0 | - | - | - | - |
| 4-point worsening | 0 | - | - | - | - |
| **By HP Interference-Quality of life** |  |  |  |  |  |
| 4-point improvement | 1 | −50.0 (-) | −50.0, −50.0, −50.0 | −50.0, −50.0 | −50.0, −50.0 |
| 3-point improvement | 3 | −51.9 (15.62) | −64.3, −57.1, −34.4 | −64.3, −34.4 | −64.3, −34.4 |
| 2-point improvement | 8 | −34.2 (16.45) | −48.9, −35.3, −23.4 | −53.1, −5.4 | −53.1, −5.4 |
| 1-point improvement | 17 | −14.0 (11.29) | −25.0, −12.5, −3.1 | −28.1, 0.0 | −32.1, 3.1 |
| No change | 18 | −7.7 (13.43) | −18.8, −3.1, 0.0 | −28.6, 9.4 | −39.3, 12.5 |
| 1-point worsening | 8 | 7.0 (9.19) | 1.6, 6.7, 12.5 | −7.1, 21.4 | −7.1, 21.4 |
| 2-point worsening | 2 | 27.0 (7.26) | 21.9, 27.0, 32.1 | 21.9, 32.1 | 21.9, 32.1 |
| 3-point worsening | 1 | 0.0 (-) | 0.0, 0.0, 0.0 | 0.0, 0.0 | 0.0, 0.0 |
| 4-point worsening | 0 | - | - | - | - |
| **By PGIS Cognitive Symptoms** |  |  |  |  |  |
| 5-point improvement | 0 | - | - | - | - |
| 4-point improvement | 1 | −50.0 (-) | −50.0, −50.0, −50.0 | −50.0, −50.0 | −50.0, −50.0 |
| 3-point improvement | 4 | −47.3 (18.04) | −58.7, −51.6, −35.9 | −64.3, −21.9 | −64.3, −21.9 |
| 2-point improvement | 9 | −21.2 (15.43) | −27.2, −18.8, −10.7 | −47.8, 0.0 | −47.8, 0.0 |
| 1-point improvement | 15 | −12.1 (21.69) | −31.3, −3.1, 3.1 | −39.3, 12.5 | −57.1, 21.4 |
| No change | 15 | −7.1 (12.63) | −18.8, −3.1, 0.0 | −21.9, 3.1 | −34.4, 17.9 |
| 1-point worsening | 10 | −8.8 (14.20) | −25.0, −5.1, 0.0 | −28.3, 8.3 | −28.6, 9.4 |
| 2-point worsening | 3 | 13.1 (16.88) | 0.0, 7.1, 32.1 | 0.0, 32.1 | 0.0, 32.1 |
| 3-point worsening | 0 | - | - | - | - |
| 4-point worsening | 1 | 21.9 (-) | 21.9, 21.9, 21.9 | 21.9, 21.9 | 21.9, 21.9 |
| 5-point worsening | 0 | - | - | - | - |
| **By CGIS Cognitive Symptoms** |  |  |  |  |  |
| 5-point improvement | 0 | - | - | - | - |
| 4-point improvement | 1 | −50.0 (-) | −50.0, −50.0, −50.0 | −50.0, −50.0 | −50.0, −50.0 |
| 3-point improvement | 1 | −53.1 (-) | −53.1, −53.1, −53.1 | −53.1, −53.1 | −53.1, −53.1 |
| 2-point improvement | 5 | −17.9 (15.37) | −21.9, −21.9, −6.3 | −39.3, 0.0 | −39.3, 0.0 |
| 1-point improvement | 16 | −17.2 (21.11) | −32.8, −8.9, −1.6 | −50.0, 0.0 | −64.3, 9.4 |
| No change | 17 | −9.0 (18.00) | −18.8, −5.4, 0.0 | −28.6, 21.4 | −47.8, 21.9 |
| 1-point worsening | 8 | 0.3 (17.28) | −10.9, 1.6, 7.1 | −25.0, 32.1 | −25.0, 32.1 |
| 2-point worsening | 2 | 15.2 (3.79) | 12.5, 15.2, 17.9 | 12.5, 17.9 | 12.5, 17.9 |
| 3-point worsening | 0 | - | - | - | - |
| 4-point worsening | 0 | - | - | - | - |
| 5-point worsening | 0 | - | - | - | - |
| **By Standard of care** |  |  |  |  |  |
| On SOC | 9 | −0.7 (12.38) | −3.1, 0.0, 0.0 | −18.8, 21.4 | −18.8, 21.4 |
| Off SOC | 49 | −15.1 (21.39) | −28.1, −10.7, 0.0 | −50.0, 7.1 | −64.3, 32.1 |
| **Distribution-based methods** |  | **Estimate** |  |  |  |
| Half-SD |  | 13.20 |  |  |  |
| SEM |  | 13.13 |  |  |  |

HPES = Hypoparathyroidism Patient Experience Scale; CGIS = clinical global impression of severity; HP = hypoparathyroidism; PGIS = patient global impression of severity; SEM = standard error of measurement; SOC = standard of care.

Note: Gold highlights the primary anchor level and blue highlights supportive anchor levels.

### **Table S11.** Phase 2 Study Interpretation of Change – HPES-Impact Psychological Well-Being

| **Score/Methods** | **Changes in HPES From Screening to Visit 3** | | | | |
| --- | --- | --- | --- | --- | --- |
| **HPES-Impact Psychological Well-Being Domain (0-100 scale)** |  |  |  |  |  |
| **Anchor-based Methods** | **n** | **Mean (SD)** | **Q1, Median, Q3** | **10th, 90th Percentile** | **Min, Max** |
| Total Sample | 59 | −9.2 (22.24) | −18.8, −6.3, 0.0 | −34.4, 9.4 | −62.5, 62.5 |
| **PRIMARY By HP Interference-Emotional well-being** |  |  |  |  |  |
| 4-point improvement | 0 | − | − | − | − |
| 3-point improvement | 5 | −43.8 (23.07) | −62.5, −56.3, −21.9 | −62.5, −15.6 | −62.5, −15.6 |
| 2-point improvement | 7 | −25.4 (17.06) | −34.4, −21.9, −9.4 | −56.3, −6.3 | −56.3, −6.3 |
| 1-point improvement | 16 | −16.0 (15.13) | −23.4, −12.5, −4.7 | −34.4, 0.0 | −56.3, 0.0 |
| No change | 25 | 0.0 (10.97) | 0.0, 0.0, 3.1 | −15.6, 9.4 | −18.8, 31.3 |
| 1-point worsening | 6 | 18.2 (25.65) | 0.0, 10.9, 31.3 | −6.3, 62.5 | −6.3, 62.5 |
| 2-point worsening | 0 | - | - | - | - |
| 3-point worsening | 0 | - | - | - | - |
| 4-point worsening | 0 | - | - | - | - |
| **By HP Interference-Quality of life** |  |  |  |  |  |
| 4-point improvement | 1 | −56.3 (-) | −56.3, −56.3, −56.3 | −56.3, −56.3 | −56.3, −56.3 |
| 3-point improvement | 3 | −44.8 (25.45) | −62.5, −56.3, −15.6 | −62.5, −15.6 | −62.5, −15.6 |
| 2-point improvement | 8 | −31.3 (18.30) | −45.3, −21.9, −18.8 | −62.5, −15.6 | −62.5, −15.6 |
| 1-point improvement | 17 | −13.1 (10.84) | −21.9, −9.4, −6.3 | −31.3, 0.0 | −34.4, 3.1 |
| No change | 19 | 0.2 (12.69) | 0.0, 0.0, 3.1 | −18.8, 21.9 | −28.1, 31.3 |
| 1-point worsening | 8 | 2.7 (7.91) | −1.6, 0.0, 6.3 | −6.3, 18.8 | −6.3, 18.8 |
| 2-point worsening | 2 | 46.9 (22.10) | 31.3, 46.9, 62.5 | 31.3, 62.5 | 31.3, 62.5 |
| 3-point worsening | 1 | 0.0 (-) | 0.0, 0.0, 0.0 | 0.0, 0.0 | 0.0, 0.0 |
| 4-point worsening | 0 | - | - | - | - |
| **By PGIS Cognitive Symptoms** |  |  |  |  |  |
| 5-point improvement | 0 | - | - | - | - |
| 4-point improvement | 1 | −56.3 (-) | −56.3, −56.3, −56.3 | −56.3, −56.3 | −56.3, −56.3 |
| 3-point improvement | 4 | −38.3 (23.71) | −56.3, −45.3, −20.3 | −56.3, −6.3 | −56.3, −6.3 |
| 2-point improvement | 9 | −19.8 (18.81) | −18.8, −18.8, −9.4 | −62.5, 0.0 | −62.5, 0.0 |
| 1-point improvement | 15 | −3.5 (12.77) | −15.6, 0.0, 3.1 | −21.9, 9.4 | −25.0, 21.9 |
| No change | 15 | −12.1 (17.55) | −21.9, −3.1, 0.0 | −31.3, 0.0 | −62.5, 3.1 |
| 1-point worsening | 10 | −5.9 (10.77) | −9.4, −3.1, 0.0 | −23.4, 4.7 | −28.1, 9.4 |
| 2-point worsening | 4 | 26.6 (28.58) | 6.3, 25.0, 46.9 | −6.3, 62.5 | −6.3, 62.5 |
| 3-point worsening | 0 | - | - | - | - |
| 4-point worsening | 1 | 31.3 (-) | 31.3, 31.3, 31.3 | 31.3, 31.3 | 31.3, 31.3 |
| 5-point worsening | 0 | - | - | - | - |
| **By CGIS Cognitive Symptoms** |  |  |  |  |  |
| 5-point improvement | 0 | - | - | - | - |
| 4-point improvement | 1 | −34.4 (-) | −34.4, −34.4, −34.4 | −34.4, −34.4 | −34.4, −34.4 |
| 3-point improvement | 1 | −56.3 (-) | −56.3, −56.3, −56.3 | −56.3, −56.3 | −56.3, −56.3 |
| 2-point improvement | 5 | −18.8 (25.10) | −15.6, −9.4, −6.3 | −62.5, 0.0 | −62.5, 0.0 |
| 1-point improvement | 16 | −17.4 (24.58) | −29.7, −14.1, 0.0 | −56.3, 9.4 | −62.5, 21.9 |
| No change | 17 | −4.8 (12.46) | −9.4, −3.1, 0.0 | −21.9, 3.1 | −21.9, 31.3 |
| 1-point worsening | 9 | 11.1 (24.01) | 0.0, 3.1, 18.8 | −18.8, 62.5 | −18.8, 62.5 |
| 2-point worsening | 2 | 0.0 (0.00) | 0.0, 0.0, 0.0 | 0.0, 0.0 | 0.0, 0.0 |
| 3-point worsening | 0 | - | - | - | - |
| 4-point worsening | 0 | - | - | - | - |
| 5-point worsening | 0 | - | - | - | - |
| **By Standard of care** |  |  |  |  |  |
| On SOC | 10 | 1.3 (11.90) | −6.3, −1.6, 3.1 | −7.8, 20.3 | −9.4, 31.3 |
| Off SOC | 49 | −11.4 (23.31) | −21.9, −6.3, 0.0 | −56.3, 9.4 | −62.5, 62.5 |
| **Distribution-based methods** |  | **Estimate** |  |  |  |
| Half-SD |  | 11.23 |  |  |  |
| SEM |  | 14.55 |  |  |  |

HPES = Hypoparathyroidism Patient Experience Scale; CGIS = clinical global impression of severity; HP = hypoparathyroidism; PGIS = patient global impression of severity; SEM = standard error of measurement; SOC = standard of care.

Note: Gold highlights the primary anchor level and blue highlights supportive anchor levels.

### **Table S12.** Phase 2 Study Interpretation of Change – HPES-Impact Social Life and Relationships

| **Score/Methods** | **Changes in HPES from Screening to Visit 3** | | | | |
| --- | --- | --- | --- | --- | --- |
| **HPES-Impact Social Life and Relationships Domain (0-100 scale)** |  |  |  |  |  |
| **Anchor-based Methods** | **n** | **Mean (SD)** | **Q1, Median, Q3** | **10th, 90th Percentile** | **Min to Max** |
| Total Sample | 59 | −7.6 (20.95) | −20.0, −5.0, 0.0 | −30.0, 12.5 | −65.0, 45.0 |
| **PRIMARY By HP Interference-Social functioning** |  |  |  |  |  |
| 4-point improvement | 0 | - | - | - | - |
| 3-point improvement | 2 | −37.5 (17.68) | −50.0, −37.5, −25.0 | −50.0, −25.0 | −50.0, −25.0 |
| 2-point improvement | 11 | −32.7 (17.52) | −50.0, −30.0, −15.0 | −55.0, −15.0 | −65.0, −10.0 |
| 1-point improvement | 14 | −8.1 (11.82) | −20.0, −5.0, 0.0 | −25.0, 6.3 | −25.0, 10.0 |
| No change | 24 | −3.6 (7.70) | −10.0, 0.0, 0.0 | −12.5, 0.0 | −25.0, 10.0 |
| 1-point worsening | 7 | 21.8 (17.95) | 0.0, 25.0, 35.0 | 0.0, 45.0 | 0.0, 45.0 |
| 2-point worsening | 1 | 35.0 (-) | 35.0, 35.0, 35.0 | 35.0, 35.0 | 35.0, 35.0 |
| 3-point worsening | 0 | - | - | - | - |
| 4-point worsening | 0 | - | - | - | - |
| **By HP Interference-Quality of life** |  |  |  |  |  |
| 4-point improvement | 1 | −30.0 (-) | −30.0, −30.0, −30.0 | −30.0, −30.0 | −30.0, −30.0 |
| 3-point improvement | 3 | −50.0 (15.00) | −65.0, −50.0, −35.0 | −65.0, −35.0 | −65.0, −35.0 |
| 2-point improvement | 8 | −29.4 (15.91) | −40.0, −27.5, −17.5 | −55.0, −10.0 | −55.0, −10.0 |
| 1-point improvement | 17 | −12.2 (10.15) | −25.0, −10.0, −5.0 | −25.0, 0.0 | −25.0, 0.0 |
| No change | 19 | 1.1 (11.52) | 0.0, 0.0, 0.0 | −10.0, 6.3 | −10.0, 45.0 |
| 1-point worsening | 8 | 10.3 (14.42) | 0.0, 10.0, 18.8 | −10.0, 35.0 | −10.0, 35.0 |
| 2-point worsening | 2 | 35.0 (0.00) | 35.0, 35.0, 35.0 | 35.0, 35.0 | 35.0, 35.0 |
| 3-point worsening | 1 | 0.0 (-) | 0.0, 0.0, 0.0 | 0.0, 0.0 | 0.0, 0.0 |
| 4-point worsening | 0 | - | - | - | - |
| **By PGIS Cognitive Symptoms** |  |  |  |  |  |
| 5-point improvement | 0 | - | - | - | - |
| 4-point improvement | 1 | −30.0 (-) | −30.0, −30.0, −30.0 | −30.0, −30.0 | −30.0, −30.0 |
| 3-point improvement | 4 | −41.3 (22.13) | −60.0, −40.0, −22.5 | −65.0, −20.0 | −65.0, −20.0 |
| 2-point improvement | 9 | −19.7 (14.71) | −25.0, −15.0, −10.0 | −50.0, 0.0 | −50.0, 0.0 |
| 1-point improvement | 15 | −7.7 (14.62) | −10.0, 0.0, 0.0 | −25.0, 0.0 | −50.0, 10.0 |
| No change | 15 | −6.6 (13.66) | −15.0, 0.0, 0.0 | −30.0, 6.3 | −35.0, 10.0 |
| 1-point worsening | 10 | −1.5 (15.10) | −10.0, 0.0, 0.0 | −17.5, 17.5 | −25.0, 35.0 |
| 2-point worsening | 4 | 29.4 (13.90) | 18.8, 30.0, 40.0 | 12.5, 45.0 | 12.5, 45.0 |
| 3-point worsening | 0 | - | - | - | - |
| 4-point worsening | 1 | 35.0 (-) | 35.0, 35.0, 35.0 | 35.0, 35.0 | 35.0, 35.0 |
| 5-point worsening | 0 | - | - | - | - |
| **By CGIS Cognitive Symptoms** |  |  |  |  |  |
| 5-point improvement | 0 | - | - | - | - |
| 4-point improvement | 1 | −20.0 (-) | −20.0, −20.0, −20.0 | −20.0, −20.0 | −20.0, −20.0 |
| 3-point improvement | 1 | −55.0 (-) | −55.0, −55.0, −55.0 | −55.0, −55.0 | −55.0, −55.0 |
| 2-point improvement | 5 | −23.0 (19.24) | −30.0, −25.0, −10.0 | −50.0, 0.0 | −50.0, 0.0 |
| 1-point improvement | 16 | −11.2 (19.44) | −25.0, 0.0, 0.0 | −35.0, 0.0 | −65.0, 6.3 |
| No change | 17 | −3.1 (16.19) | −12.5, −5.0, 0.0 | −25.0, 25.0 | −30.0, 35.0 |
| 1-point worsening | 9 | 9.2 (24.24) | −10.0, 0.0, 35.0 | −25.0, 45.0 | −25.0, 45.0 |
| 2-point worsening | 2 | 5.0 (7.07) | 0.0, 5.0, 10.0 | 0.0, 10.0 | 0.0, 10.0 |
| 3-point worsening | 0 | - | - | - | - |
| 4-point worsening | 0 | - | - | - | - |
| 5-point worsening | 0 | - | - | - | - |
| **By Standard of care** |  |  |  |  |  |
| On SOC | 10 | 3.8 (19.76) | −10.0, 0.0, 10.0 | −18.8, 35.0 | −25.0, 45.0 |
| Off SOC | 49 | −9.9 (20.61) | −25.0, −5.0, 0.0 | −35.0, 10.0 | −65.0, 35.0 |
| **Distribution-based methods** |  | **Estimate** |  |  |  |
| Half-SD |  | 11.11 |  |  |  |
| SEM |  | 13.57 |  |  |  |

HPES = Hypoparathyroidism Patient Experience Scale; CGIS = clinical global impression of severity; HP = hypoparathyroidism; PGIS = patient global impression of severity; SEM = standard error of measurement; SOC = standard of care.

Note: Gold highlights the primary anchor level and blue highlights supportive anchor levels.

### **Fig. S7.** Cumulative Distribution Function of Change in HPES Impact Total Score from Screening to Visit 3, by HP interference-Quality of Life


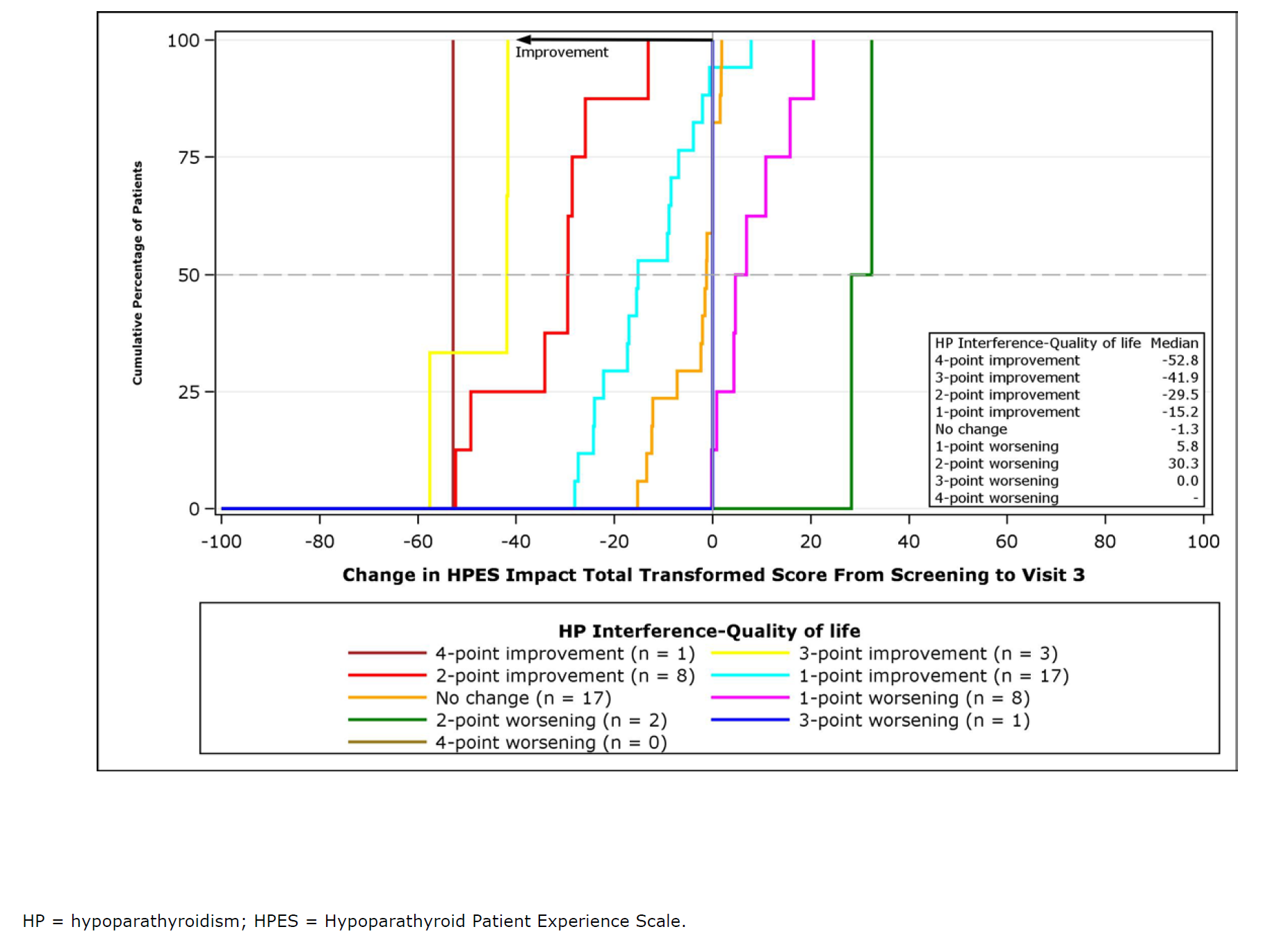


### **Fig. S8.** Probability Density Function of Change in HPES Impact Total Score from Screening to Visit 3, by HP interference- Quality of Life


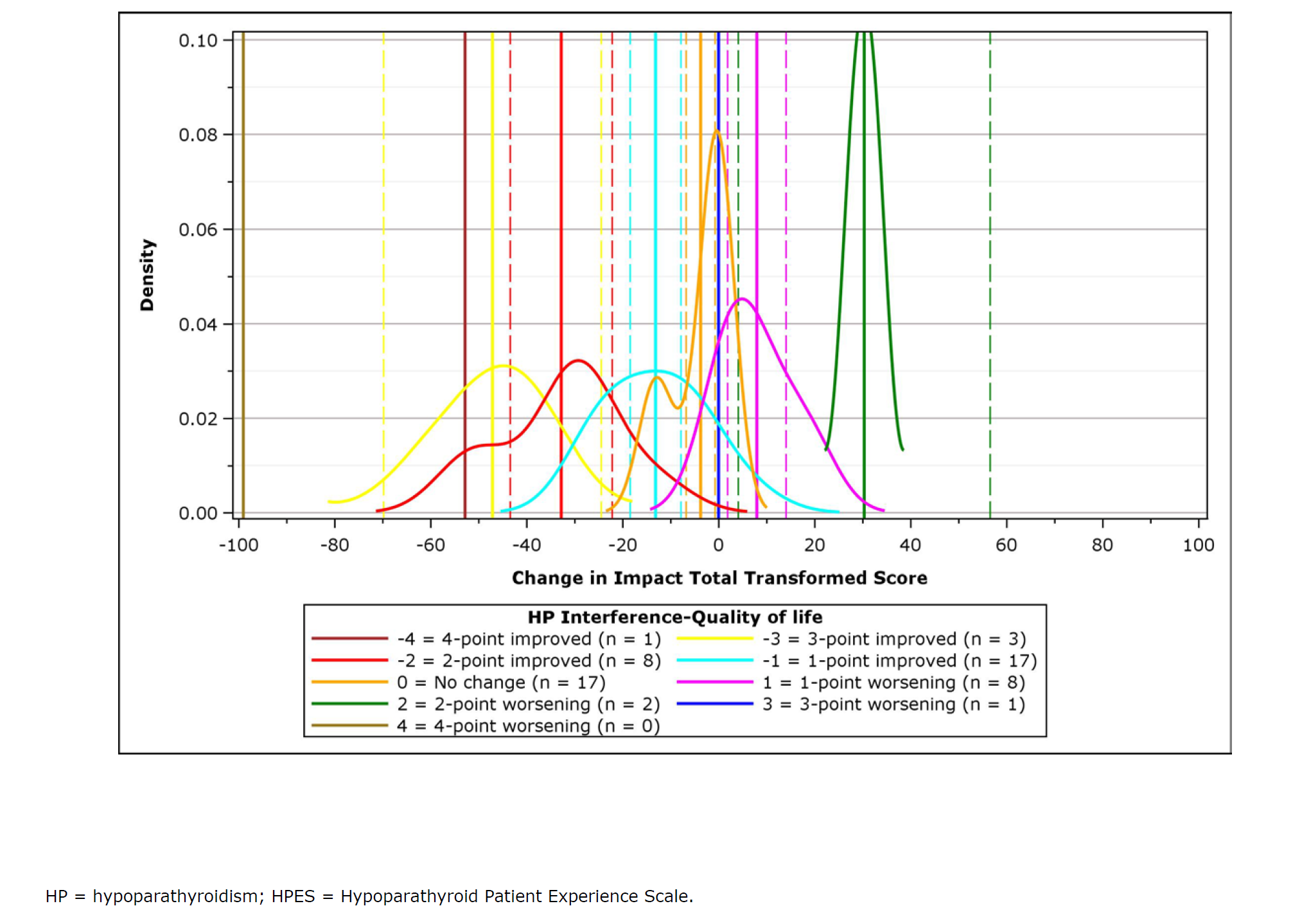


### **Fig. S9.** Cumulative Distribution Function of Change in HPES Impact Physical Functioning Score from Screening to Visit 3, by HP interference-Physical functioning


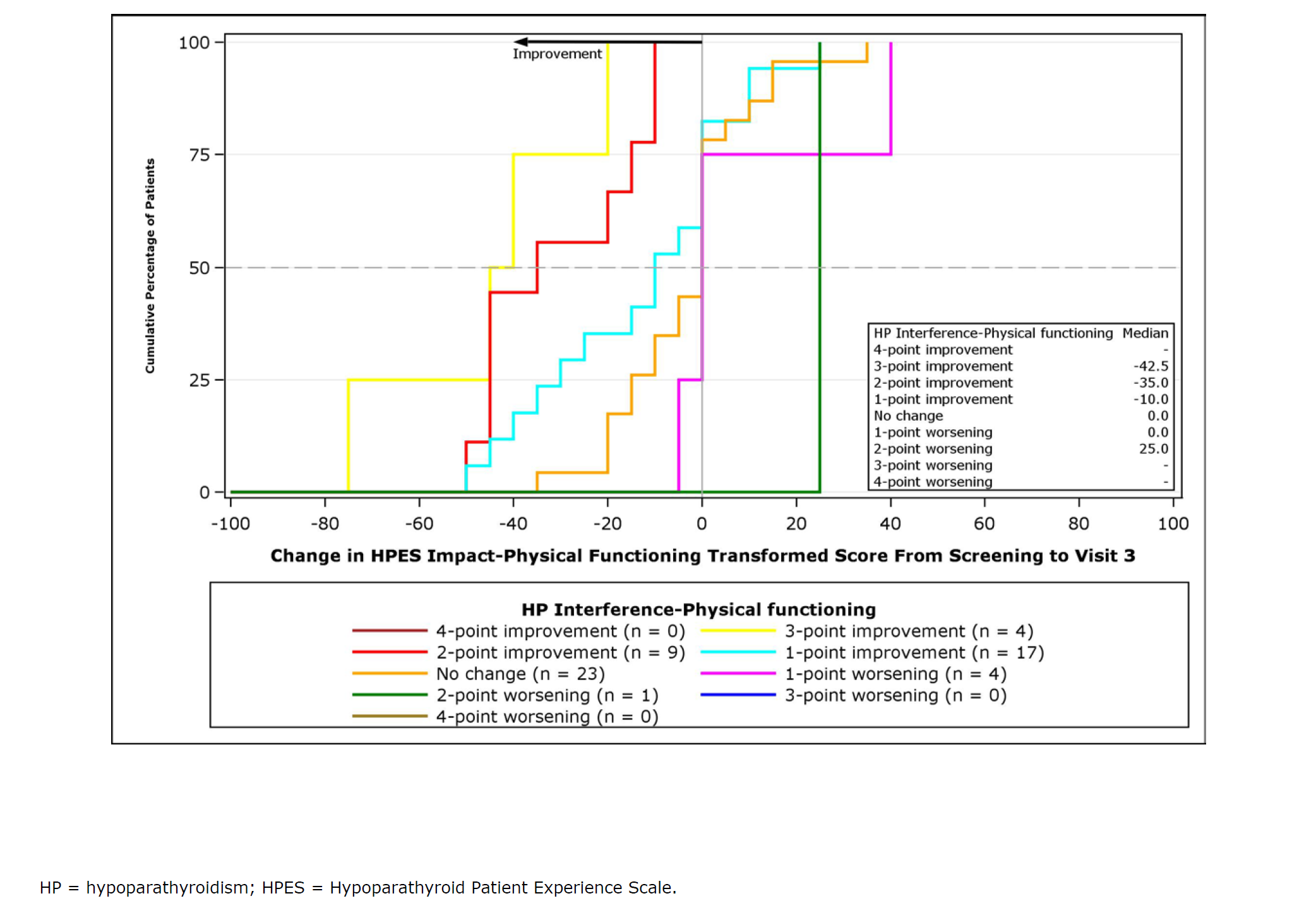


### **Fig. S10.** Probability Density Function of Change in HPES Impact Physical Functioning Score from Screening to visit 3, by HP interference-Physical functioning


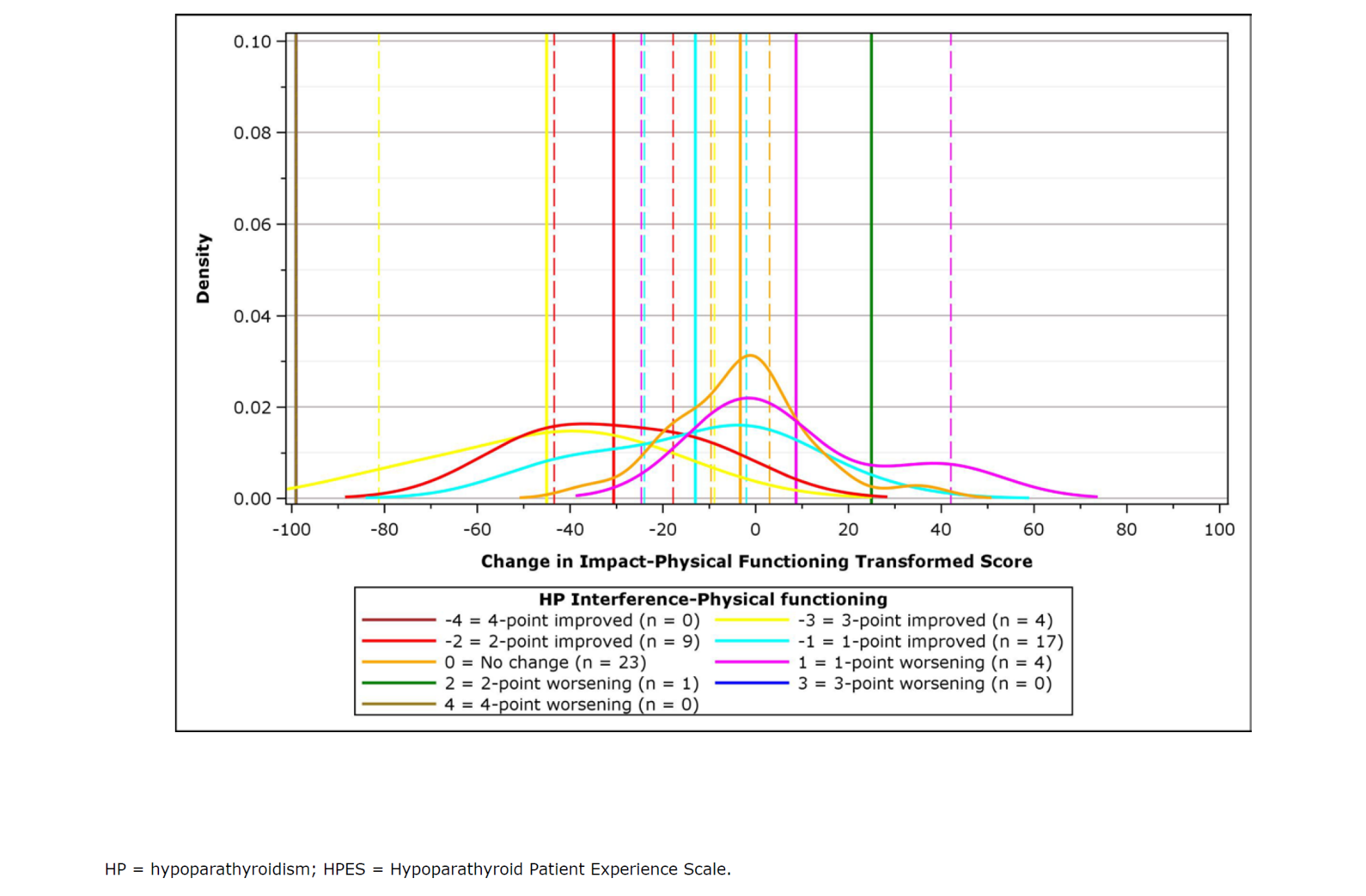


### **Fig. S11.** Cumulative Distribution Function of Change in HPES Impact Daily Life Score from Screening to Visit 3, by HP interference–Daily Functioning


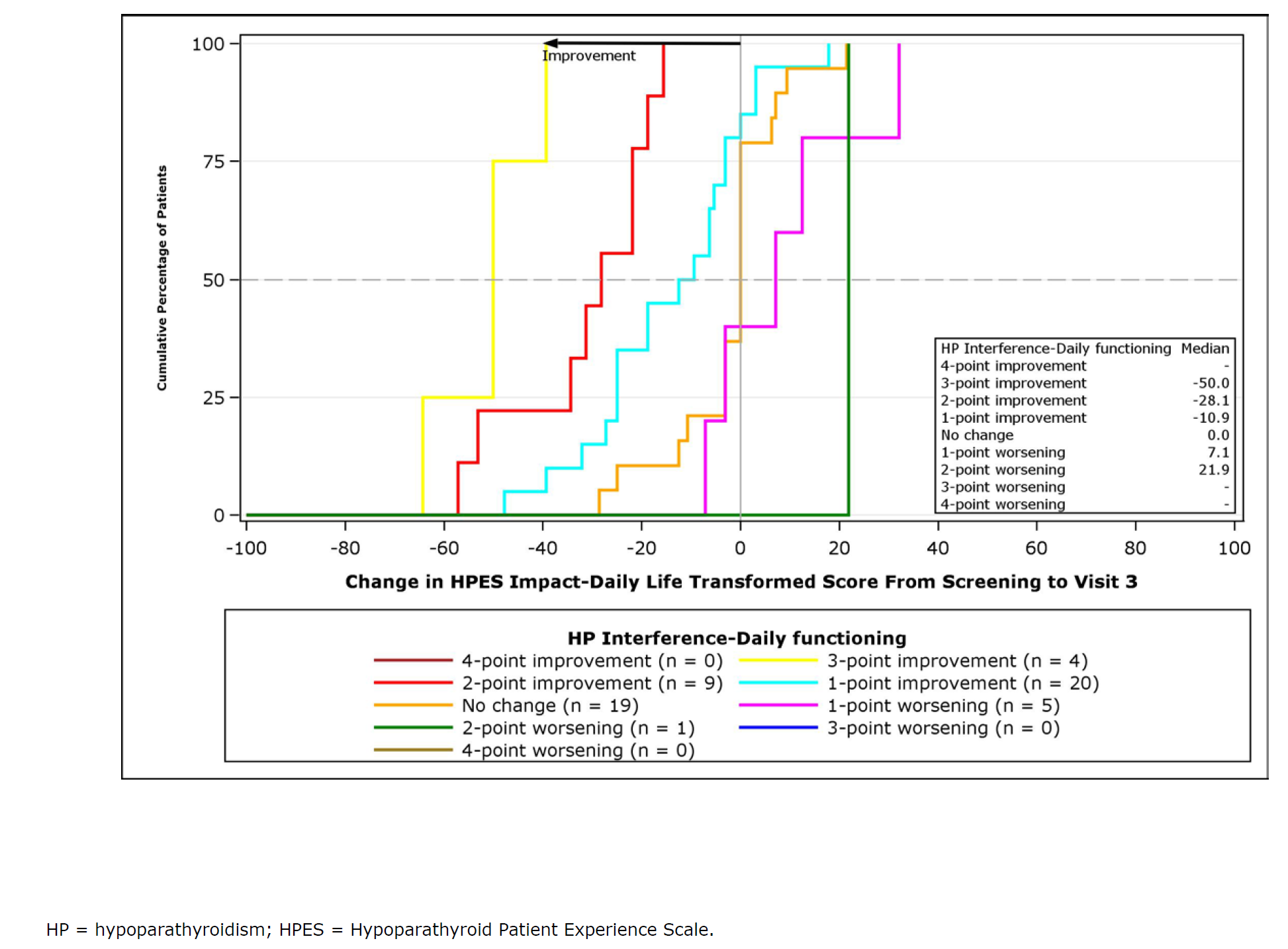


### **Fig. S12.** Probability Density Function of Change in HPES Impact Daily Life Score from Screening to Visit 3, HP Interference–Daily Functioning


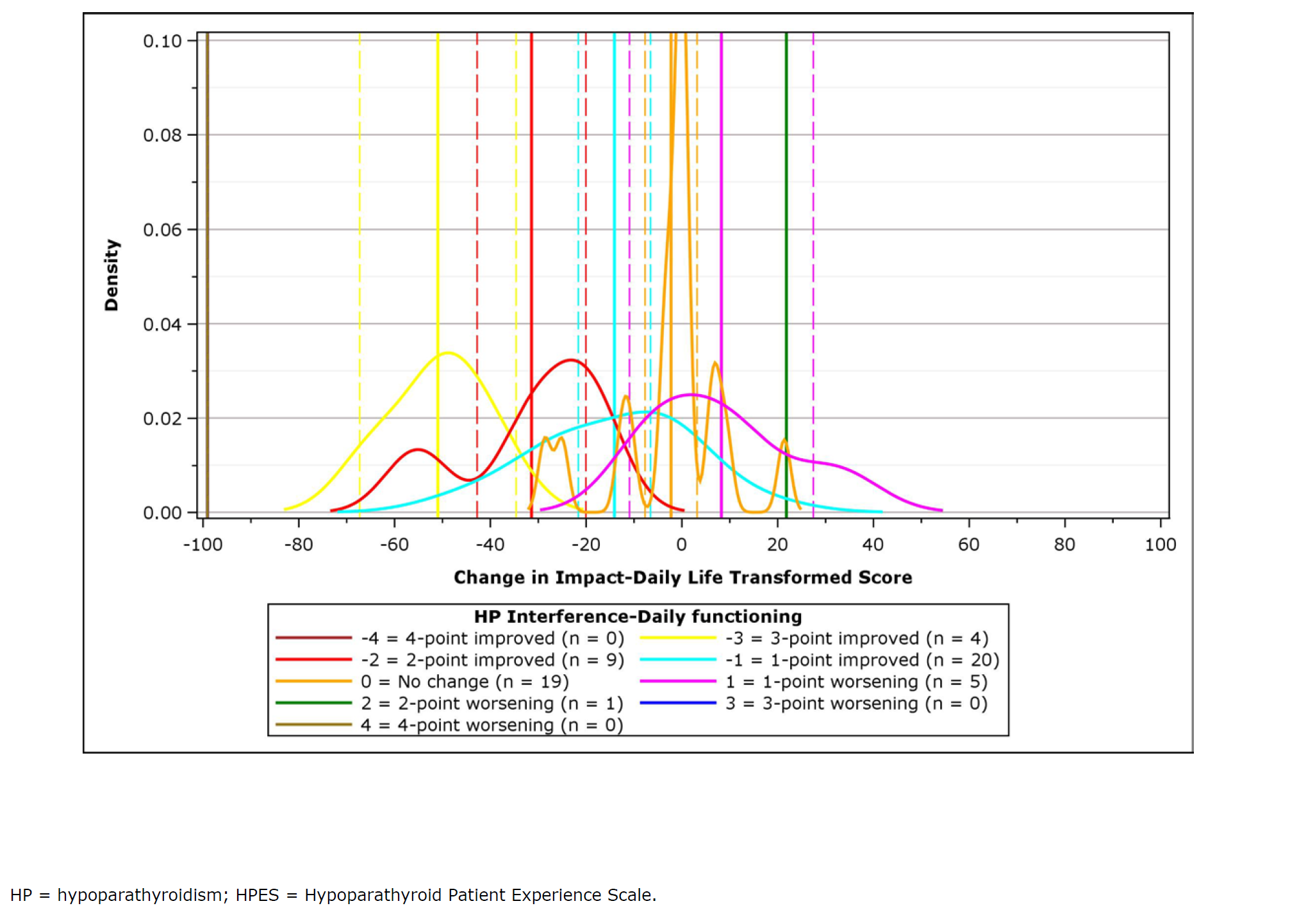


**Fig. S13.** Cumulative Distribution Function of Change in HPES Impact Psychological Well-Being Score from Screening to Visit 3, by HP interference-Emotional Well-being**
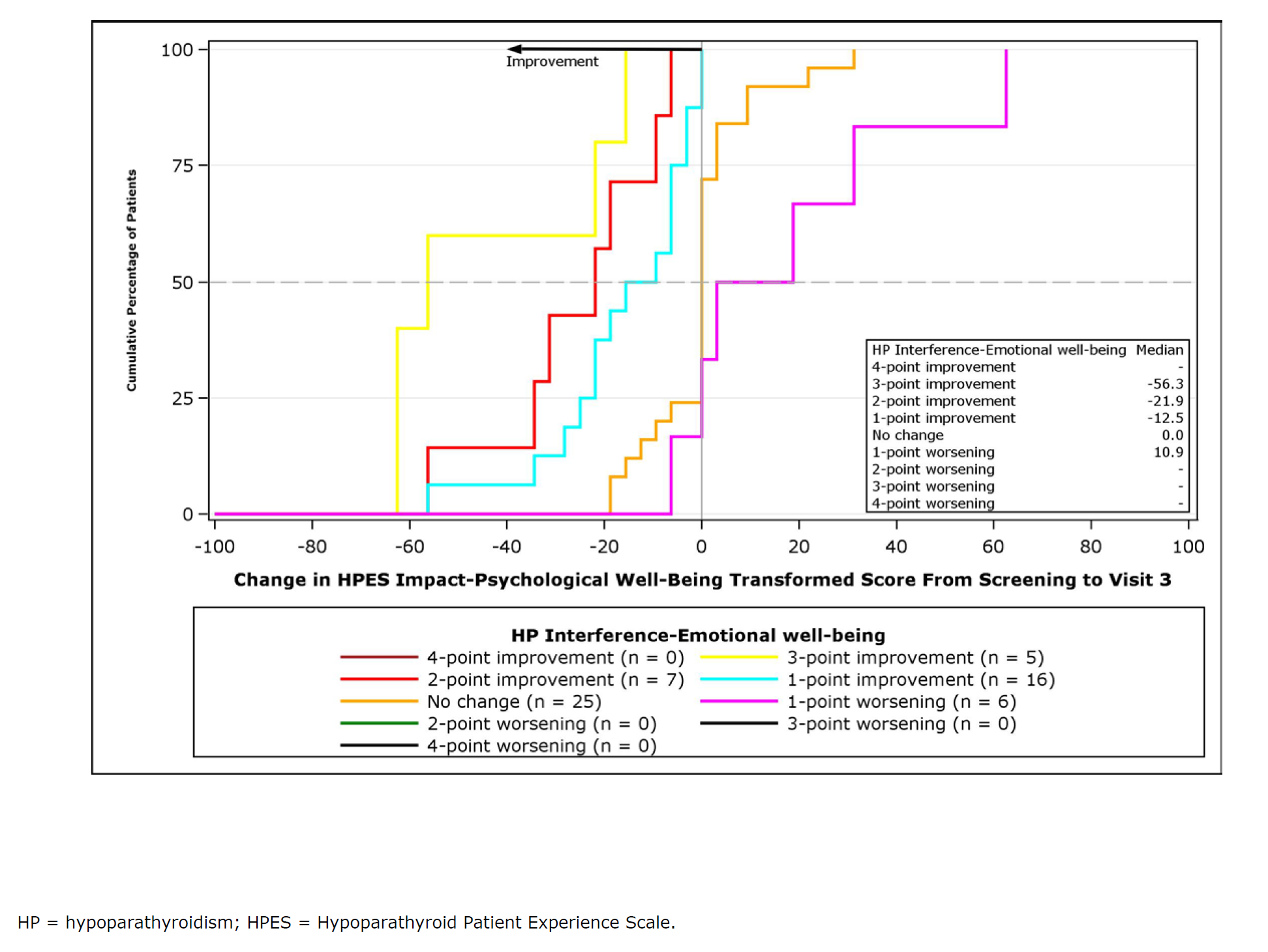
**

### **Fig. S14.** Probability Density Function of Change in HPES Impact Psychological Well-Being Score from Screening to Visit 3, by HP interference-Emotional Well-being


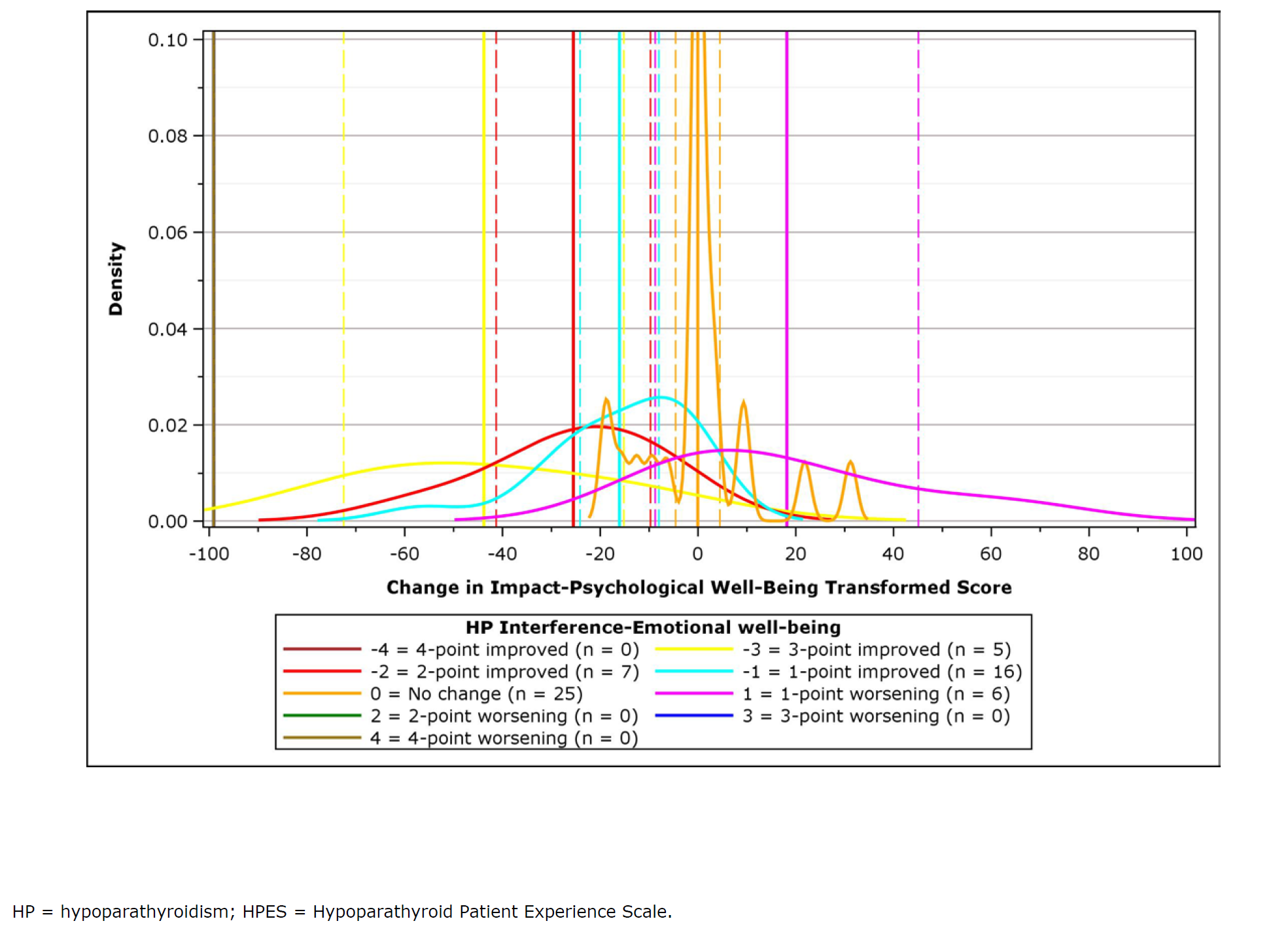


### **Fig. S15.** Cumulative Distribution Function of Change in HPES Impact Social Life and Relationships Score from Screening to Visit 3, by HP interference-Social Functioning

**
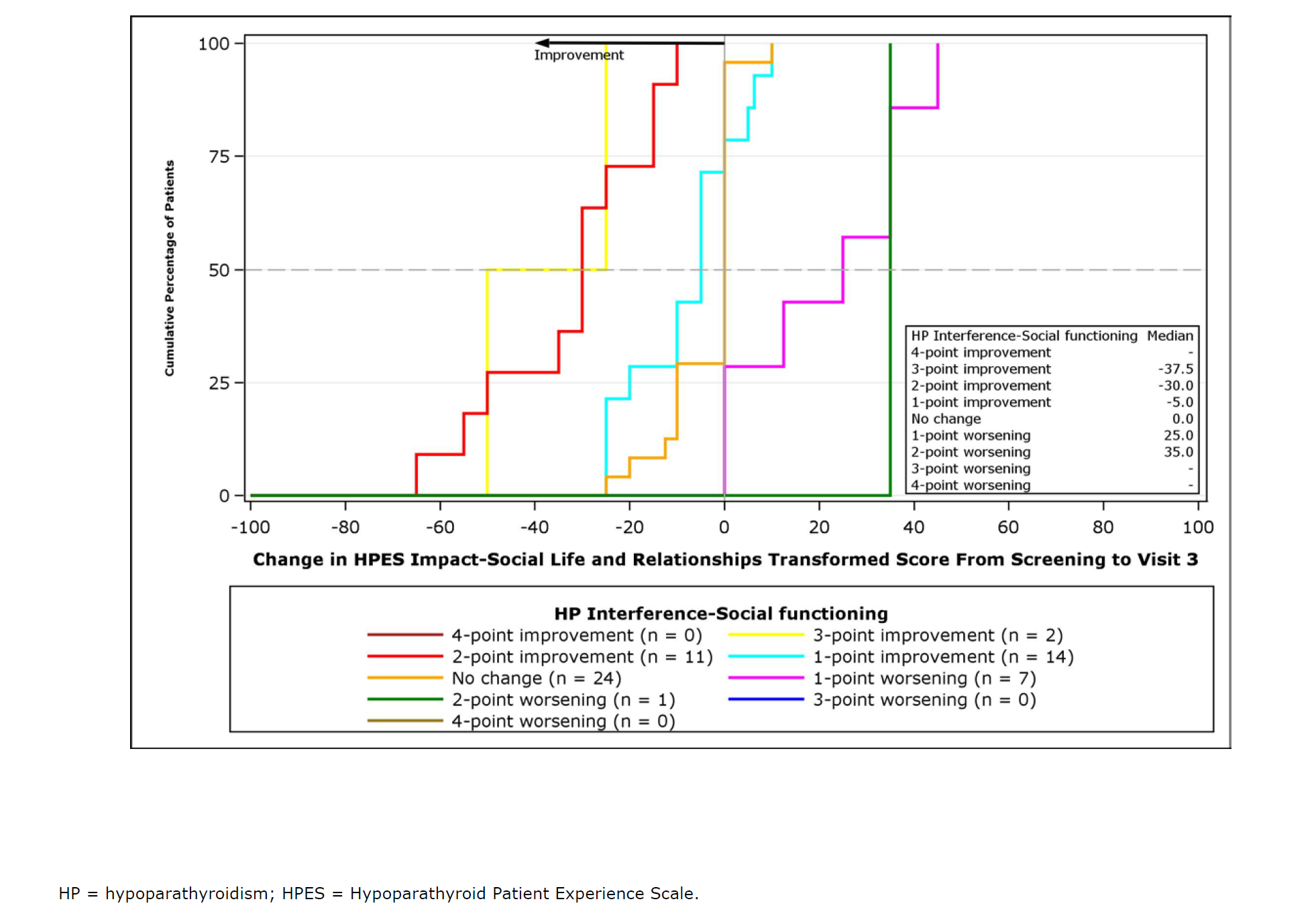
**

### **Fig. S16.** Probability Density Function of Change in HPES Impact Social Life and Relationships Score from Screening to Visit 3, by HP interference-Social Functioning


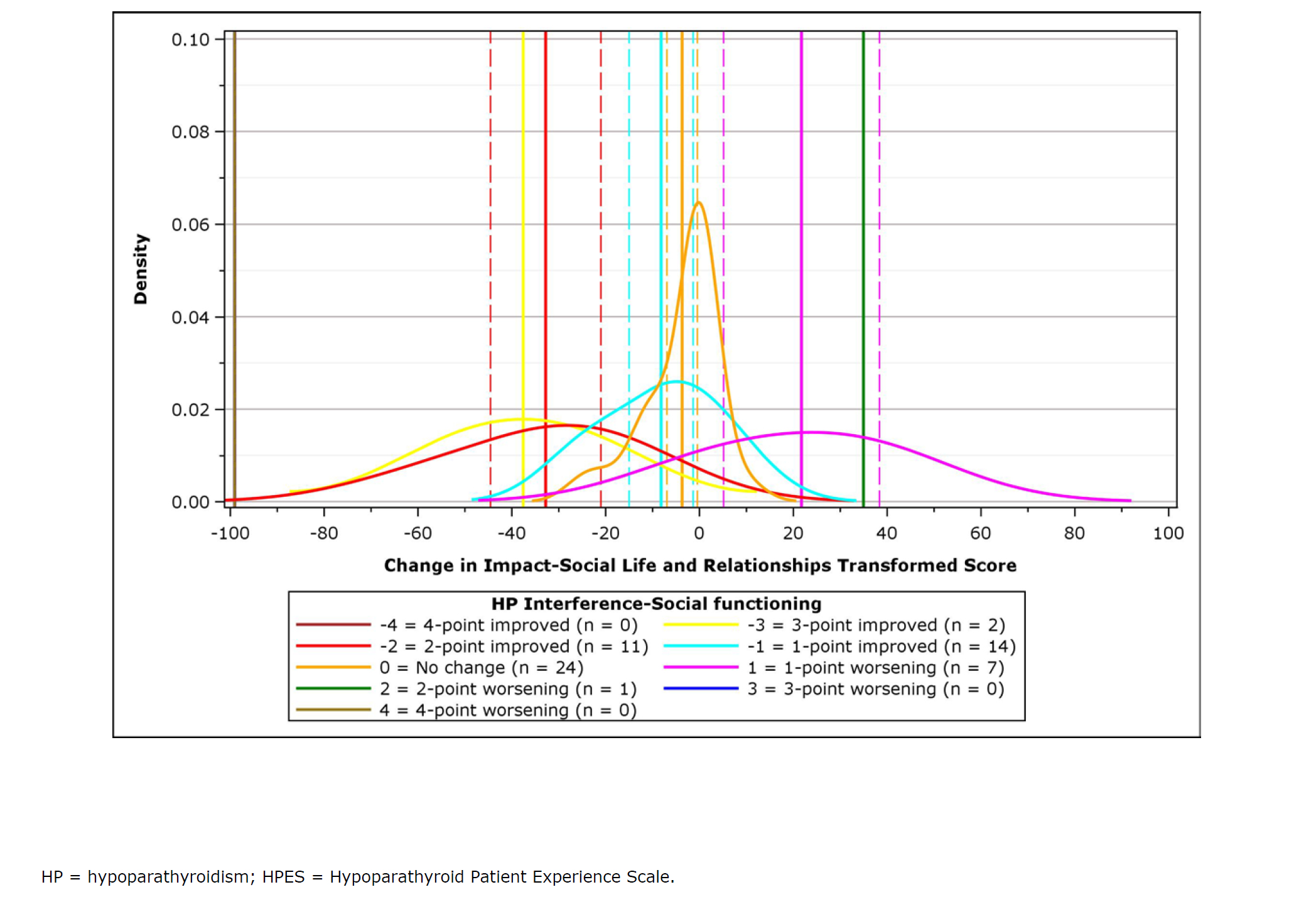

Supplement: Supplementary file 2 — Additional file 2. [file 41687_2021_320_MOESM2_ESM.docx]
